# A graph-convolutional neural network model for the prediction of chemical reactivity†
†Electronic supplementary information (ESI) available: Additional model and dataset details, results, discussion, and ref. 38 and 39. See DOI: 10.1039/c8sc04228d


**DOI:** 10.1039/c8sc04228d

**Published:** 2018-11-26

**Authors:** Connor W. Coley, Wengong Jin, Luke Rogers, Timothy F. Jamison, Tommi S. Jaakkola, William H. Green, Regina Barzilay, Klavs F. Jensen

**Affiliations:** a Department of Chemical Engineering , Massachusetts Institute of Technology , 77 Massachusetts Avenue , Cambridge , MA 02139 , USA . Email: kfjensen@mit.edu; b Computer Science and Artificial Intelligence Laboratory , Massachusetts Institute of Technology , 77 Massachusetts Avenue , Cambridge , MA 02139 , USA . Email: regina@csail.mit.edu; c Department of Chemistry , Massachusetts Institute of Technology , 77 Massachusetts Avenue , Cambridge , MA 02139 , USA

## Abstract

We present a supervised learning approach to predict the products of organic reactions given their reactants, reagents, and solvent(s). The prediction task is factored into two stages comparable to manual expert approaches: considering possible sites of reactivity and evaluating their relative likelihoods. By training on hundreds of thousands of reaction precedents covering a broad range of reaction types from the patent literature, the neural model makes informed predictions of chemical reactivity. The model predicts the major product correctly over 85% of the time requiring around 100 ms per example, a significantly higher accuracy than achieved by previous machine learning approaches, and performs on par with expert chemists with years of formal training. We gain additional insight into predictions *via* the design of the neural model, revealing an understanding of chemistry qualitatively consistent with manual approaches.

## Abstract 2

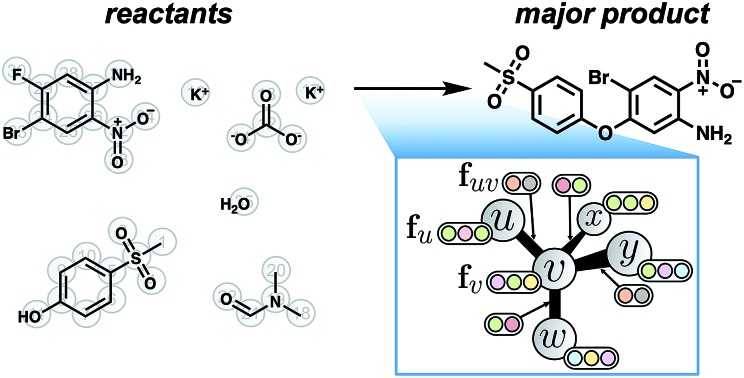
We present a supervised learning approach to predict the products of organic reactions given their reactants, reagents, and solvent(s).

## Introduction

The prediction of reaction outcomes is a fundamental exercise in chemistry. The ability to anticipate reaction products correctly enables chemists to realize more quickly the chemical compounds that they desire and that form the basis of pharmaceutical, electronic, optical, and mechanical applications. A successful computational approach to this as-yet manual task could confirm chemist intuition about different modes of reactivity, enumerate potential side products preceding structural elucidation of a complex mixture, and validate retrosynthetic suggestions from a computer-aided synthesis planning program to increase the confidence of experimental success.

As a significant step toward this ultimate goal of reaction evaluation, we address the task of predicting the major products of organic reactions based on the reactant, reagent, and solvent species. The methodology we describe below is directly applicable to the automated identification of not just major products, but all species in a mixture of products. This fulfills an additional need for impurity identification and quantification during process development, particularly in the context of drug substance manufacturing where it is essential to understand the exact composition of crude product mixtures. Reaction evaluation also play central role in the hypothesize-make-measure iterative cycle underlying small molecule discovery. Small-scale high throughput experimentation has tremendously accelerated chemical synthesis,^1^,^2^ but analysis and interpretation of results has lagged behind. A recent Merck study^3^ employed MALDI-TOF MS for rapid online analysis or 1536 well plates in just 10 minutes. Yet with these high throughput analyses, one must specify which masses to quantify or inspect results manually – in the case of the Merck study, there were “nearly 400” distinct mass peaks to extract.

There is a rich history of computer-assistance in chemical synthesis^4^–^6^ including this task of reaction prediction. In 1980, Jorgensen and coworkers introduced Computer Assisted Mechanistic Evaluation of Organic Reactions (CAMEO).^7^ This and other early approaches, including EROS,^8^ IGOR,^9^ SOPHIA,^10^ and Robia^11^ use expert heuristics to define possible mechanistic reactions. What most of these approaches have in common, advocated particularly strongly by Ugi,^9^ is the desire to enable predictions of novel chemistry, *i.e.*, reactions that do not correspond to a previously known, codifiable reaction template. However, none achieved broad use within the chemistry community.

Major developments in machine learning and data availability have enabled new approaches to this problem.^12^ For specific reaction families with sufficiently detailed reaction condition data, machine learning can be applied to the quantitative prediction of yield.^13^ Ideally, however, models could be trained on historical reaction data to predict a broad range of reaction families. One way to do so is by combining the traditional use of reaction templates in cheminformatics with machine learning, either by learning to select relevant reaction rules from a template library^14^,^15^ or by learning to rank template-generated products.^16^ Reaction templates are the classic approach to codifying the “rules” of chemistry,^17^–^20^ whose use dates back to Corey's synthesis planning program Logic and Heuristics Applied to Synthetic Analysis (LHASA).^21^ Presently, decades later, reaction template approaches continue to find extensive applications in computer-aided synthesis planning.^12^,^20^,^22^ However, while reaction rules are suitable for interpolating known chemistry to novel substrates, they leave no opportunity to describe reactions with even minor structural differences at the reaction center. Other approaches have involved learning to propose mechanistic or pseudo-mechanistic reaction steps,^23^–^26^ but these require human annotations or heuristically-generated mechanisms in addition to published experimental results. At the other extreme, one can neglect all chemical domain knowledge and use off-the-shelf machine translation models to generate products directly from reactants.^27^,^28^ Here, we describe a chemically-informed model that incorporates domain expertise through its architecture.

**Fig. 1:**
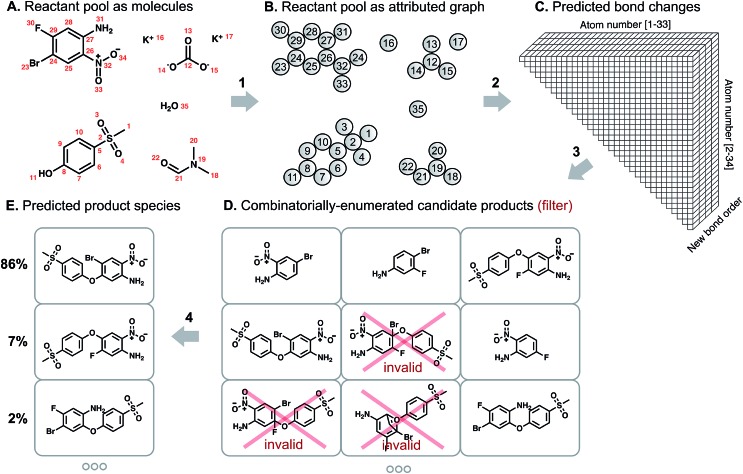
Schematic of the approach to predicting reaction products. We represent the (A) pool of reactant molecules as a (B) attributed graph. A graph convolutional neural network learns to calculate (C) likelihood scores for each bond change between each atom pair. The most likely changes are used to perform a focused, ranked enumeration of (D) candidate products, which are filtered by chemical valence rules. These candidates are then rescored by another graph convolutional network to yield (E) a probability distribution over predicted product species.

Our overall model structure (Fig. 1) is designed to reflect how expert chemists approach the same task. First, we learn to identify reactive sites that are most likely to undergo a change in connectivity – this parallels the identification of reactive functional groups and consideration of how they might react, but without codifying rigid rules about functional group decomposition (Fig. 1: arrow 2). Next, we perform a focused enumeration of products that could result from those interactions subject to chemical valence rules (Fig. 1: arrow 3). We learn to rank those candidates – determining what modes of reactivity are most likely, as would a chemist – to produce the final prediction of major products (Fig. 1: arrow 4). By dividing the prediction task into these two stages of reactivity perception and outcome scoring, we can gain insight into the neural model's suggestions and find qualitative alignment with how chemists analyze organic reactivity. The key to the success of our approach is learning a representation of molecules that captures properties relevant to reactivity. We use a graph-based representation of reactant species to propose changes in bond order, introduced in a recent conference publication.^29^ Graphs provide a natural way of describing molecular structure; nodes correspond to atoms, and edges to bonds. Indeed, graph theoretical approaches have been used to analyze various aspects of chemical systems^30^ and even for the representation of reactions themselves.^31^ As we show below, the formalization of predicting reaction outcomes as predicting graph edits – which bonds are broken, which are formed – enables the design and application of graph convolutional models that can begin to understand chemical reactivity.

A quantitative analysis of model performance shows an accuracy of over 85.6%, 5.3% higher than the previous state-of-the-art, and performance on par with human experts for this complex prediction task. Predictions are made on the order of 100 ms per example on a single consumer GPU, enabling its application to virtual screening pipelines and computer-assisted retrosynthesis workflows. More importantly however, the model provides the capacity for collaborative interaction with human chemists through its interpretability. Despite the utility of high-performing black box models, we argue that understanding predictions is equally important. Reaction prediction models that operate on the level of mechanistic steps offer a clear parallel to how human chemists rationalize how reactions proceed.^23^–^26^ The Baldi group's ReactionPredictor learns mechanisms from expert-encoded rules as a supervised learning problem^24^ and Bradshaw *et al.*'s ELECTRO model reproduces pseudo-mechanistic steps as defined by an expert-encoded heuristic function.^26^ Neither of these approaches enables models to develop their own justifications for predictions, and neither demonstrates perception of reagent effects. Schwaller *et al.*'s translation model can illustrate which reactant tokens inform which product tokens,^28^ which is useful for predicting atom-to-atom mapping, but does not reveal chemical understanding and is not aligned with how humans describe chemical reactivity.

## Results

### Perceiving likely modes of reactivity

We describe a reaction as a set of changes in bond order in a collection of reactant molecules (Fig. 1A). More formally, we treat these reactant molecules as a single molecular graph where nodes and edges describe atoms and bonds, respectively (Fig. 1B). Reactions are thus a set of graph edits where the edges (or lack of edges) between two or more nodes are changed. One aspect of our approach is the fact that for most organic reactions in this data set, the reaction center – the set of nodes and edges undergoing a change in connectivity – consists of a relatively small number of atoms and typically only up to 5 bonds (Table S1†). To be able to describe certain types of reactions not represented in this data set (*e.g.*, cascade reactions involving many mechanistic steps occurring at many atoms throughout the molecule), this observation would need to be revisited.

**Fig. 2:**
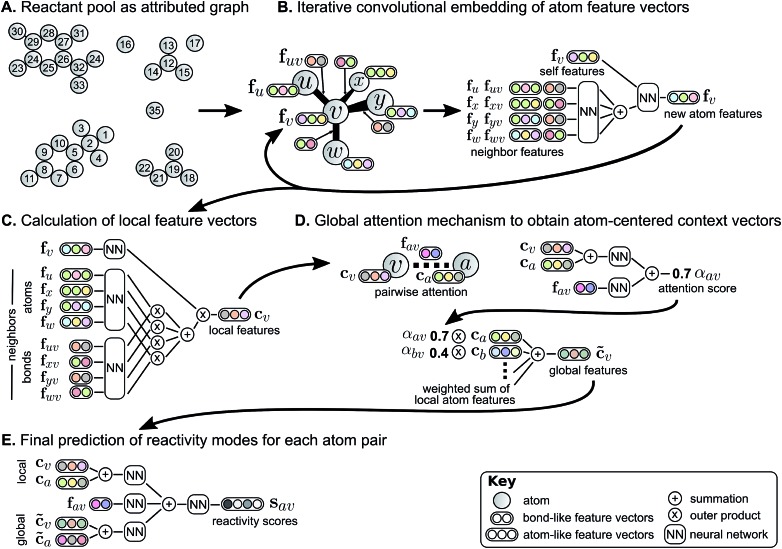
Weisfeiler-Lehman Network (WLN) model for predicting likely changes in bond order between every pair of atoms. Starting from an (A) attributed graph representation of molecules, we (B) iteratively update feature vectors describing each atom by incorporating neighboring atoms' information. After multiple iterations of this embedding, (C) a local feature vector is calculated for each atom based on its updated representation and those of its neighbors. To account for the effects of disconnected atoms such as reagents, (D) a global attention mechanism produces a context vector for each atom as a learned, weighted combination of all other atoms' local features. Finally, (E) a combination of local features and context vectors are used to predict the likelihood of bond changes for each pair of atoms.

As the first step in predicting reaction outcomes, we predict the most likely changes in connectivity: the sets of (atom, atom, new bond order) changes that describe the difference between the reactant molecules and the major product. We train a Weisfeiler-Lehman Network (WLN),^32^ a type of graph convolutional neural network, to analyze the reactant graph and predict the likelihood of each (atom, atom) pair to change to each new bond order, including a 0th order bond, *i.e.*, no bond (Fig. 1C). The WLN workflow is depicted in Fig. 2 and is described in the following paragraph. Mathematical details can be found in the ESI.†

The WLN starts with, as input, the reactant graph. Atoms are featurized by their atomic number, formal charge, degree of connectivity, explicit and implicit valence, and aromaticity. Bonds are featurized solely by their bond order and ring status. We forgo more complex atom- and bond-level descriptors (*e.g.*, partial charge estimates, surface area contributions) because these can be learned implicitly from the molecular structure and, empirically, do not improve performance. A local embedding iteratively updates atom-level representations by incorporating information from adjacent atoms, processed by a parameterized neural network. To account for the effects of distant atoms, *e.g.*, activating reagents, a global attention mechanism is used whereby all atoms in the reactant graph attend to (“look at”) all other atoms; a global context vector for each atom is based on contributions from the representations of all atoms weighted by the strength of this attention. Pairwise sums of these learned atom-level representations, both from the local atomic environment and from the influence of all other species, are used to calculate the likelihood scores. The model is trained to score the true (recorded) graph edits highly using a sigmoid cross entropy loss function.

The combinatorial nature of candidate product enumeration is drastically simplified by restricting our enumeration to only draw from the most likely *K* bond changes. By using up to 5 unique bond changes to generate each candidate outcome – a decision based on the empirically-low frequency of reactions involving more than 5 simultaneous bond changes (Table S1†) – the number of candidates is bounded by1
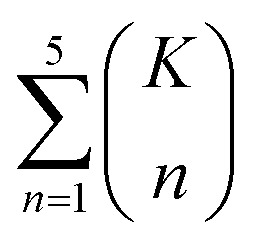
where 
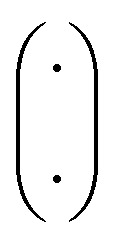
 is the binomial coefficient. Valence and connectivity constraints substantially reduce the number of valid candidates produced by this enumeration (Fig. 1D).

**Fig. 3:**
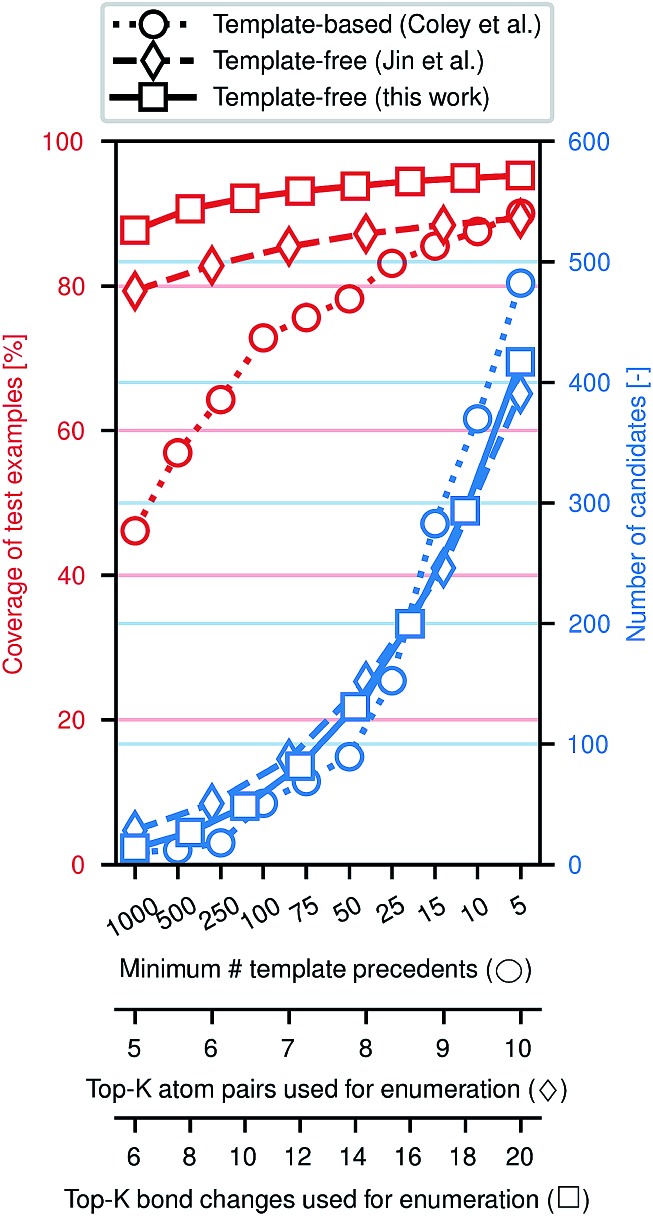
Performance of candidate enumeration approaches. The balance of generality and specified of can be tuned through different parameters for each method; *x*-axes have been scaled to approximately align the number of candidates per reaction example. Learning likely modes of reactivity increases the likelihood of including the true major product in the set of candidates.

Fig. 3 illustrates the efficacy of this approach. Using the same preprocessed training, validation, and testing sets of *ca.* 410k, 30k, and 40k reactions from the United States Patent and Trademark Office (USPTO)^33^ used previously,^28^,^29^ we evaluate how frequently the true (recorded) product of a reaction is included among the enumerated candidates, approximately normalized to the number of candidates. While an exhaustive enumeration of all possible bond changes guarantees 100% coverage, the subsequent problem of selecting the most likely outcome would be intractable. In this work, the parameter *K* can be tuned to simultaneously increase the coverage and increase the number of candidates; similar tuning is possible for the comparative template-based^16^ and graph-based^29^ enumeration strategies. In a template-based approach, one generally truncates the template library by only including templates that were derived from a certain minimum number of precedent reactions; a higher minimum threshold produces a template set covering more common reaction types.

### Evaluating candidate reaction outcomes

The set of valid product structures resulting from the focused enumeration requires additional evaluation and ranking to produce the final model prediction (Fig. 1E). We have previously treated the problem of ranking candidate products as an isolated task,^16^,^29^ yet this fails to utilize a key aspect of the enumeration approach: candidate outcomes produced by combinations of more likely bond changes are themselves more likely to be the true outcome. The quantitative scores for each bond change when perceiving likely modes of reactivity provides an initial ranking of candidate reaction outcomes. The remaining evaluation task serves to refine these preliminary rankings, now taking into account the likelihood of each set of bond changes.

The Weisfeiler-Lehman Difference Network (WLDN) used for ranking refinement is conceptually similar to the WLN (details in the ESI†). Reactants and candidate outcomes are embedded as attributed graphs to obtain local atom-level representations. For each candidate, a numerical reaction representation is calculated based on the differences in atom representations between that candidate's atoms and the reactants' atoms. The overall candidate reaction outcome score is produced by processing that reaction representation through a final neural network layer and adding it to the preliminary score obtained by summing the bond change likelihoods as perceived by the WLN. The model is trained to score the true candidate outcome most highly using a softmax cross entropy loss function. The bond changes corresponding to the top combinations are imposed on the reactant molecules to yield the predicted product molecules, which are then canonicalized in terms of their SMILES^34^ representation using RDKit.^35^

Quantitative evaluation is performed on the same atom-mapped data set of 410k/30k/40k reactions from the USPTO to enable comparison to 29 and 28. Statistics for this data set are shown in Fig. S5–S9.† For consistency, we explicitly exclude reactant species not contributing heavy atoms to the recorded products in the enumeration; that is, the model is made aware of which species are non-reactants. An exact match in product SMILES is required for a prediction to be considered correct. The performance comparison is shown in Table 1.

**Table 1:** Overall performance in reaction prediction. |*θ*| denotes the approximate size of the model (number of trainable parameters). Because all methods produce a ranked list of candidate outcomes, performance is reported in terms of top-1, 2, 3, and 5 accuracies

Method	|*θ*|	Top-1 [%]	Top-2 [%]	Top-3 [%]	Top-5 [%]
WLN/WLDN^29^	3.2 M	79.6	—	87.7	89.2
Sequence-to-sequence^28^	30 M^*a*^	80.3	84.7	86.2	87.5
This work	2.6 M	**85.6**	**90.5**	**92.8**	**93.4**

^*a*^Estimated.

The new model offers a substantial improvement in accuracy over the state of the art on this data set. In particular, the top-1, top-2, and top-3 accuracies are each over 5% higher, a significant reduction of the probability of mispredicting the major product. Comparisons to the data subset of Schwaller *et al.*^28^ (Table S2†) and Bradshaw *et al.*^26^ (Table S3†), can be found in the ESI.† Predictions are made in *ca.* 100 ms per example using a single Titan X GPU; a more detailed description of computational cost in terms of wall times for training and testing can be found in the ESI.†

**Fig. 4:**
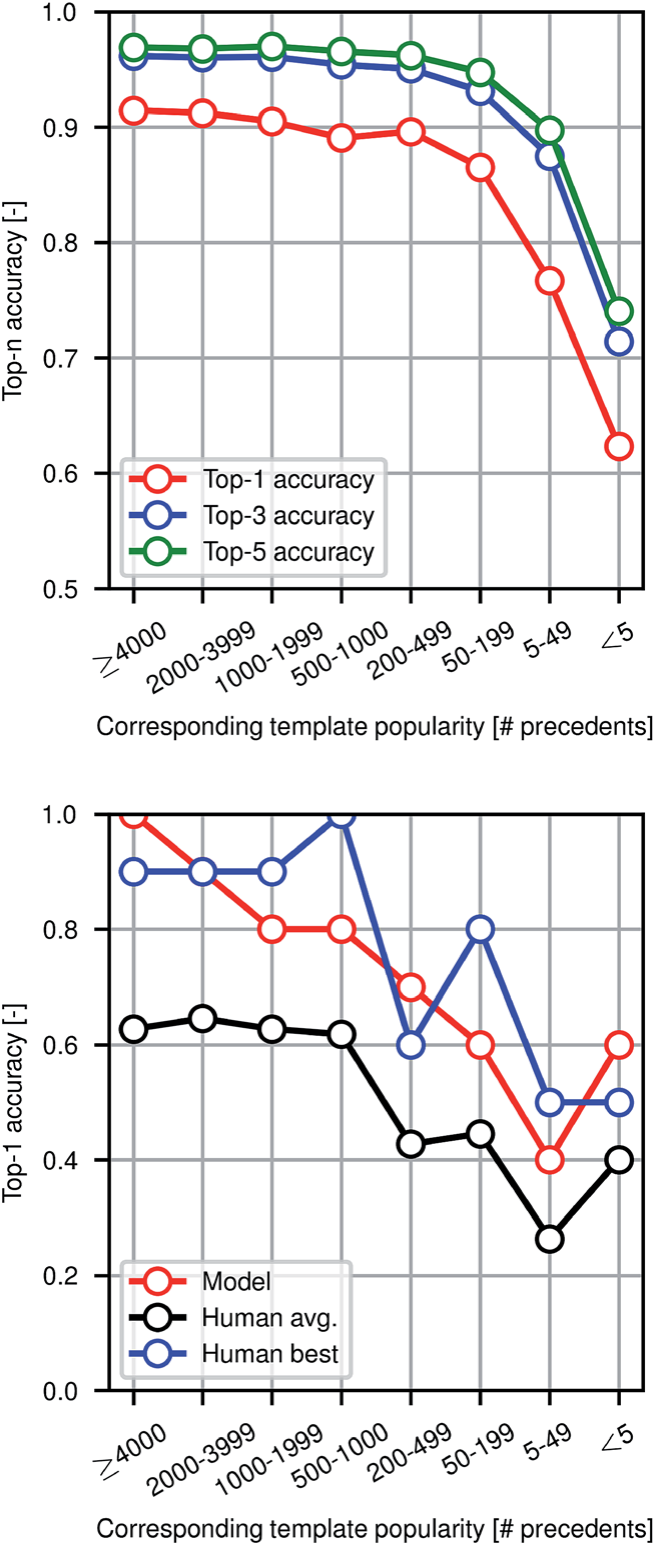
Performance in reaction prediction by the model for the entire test set of 40 000 reactions (top) and for the human benchmarking set of 80 reactions only in comparison to eleven human participants (bottom) as a function of reaction rarity, where rarity refers to the popularity of the corresponding reaction template extracted from the training set.

Fig. 4 shows model prediction performance broken down by the rarity of each reaction in the test set as measured by the popularity of the corresponding reaction template extracted from the training set. More common chemistries are empirically easier to predict, as one would expect from a data-driven approach to model reactivity. Reactions for which the corresponding template has fewer than 5 precedents (or none at all) are still predicted with >60% top-1 accuracy by the graph-based model, demonstrating its ability to generalize to previously unseen structural transformations.

### Human benchmarking

To evaluate the difficulty of this prediction task for human experts, we asked eleven human participants to write the likely major products for 80 reaction examples from the test set. The 80 total questions have been divided into 8 categories of 10 randomly-selected questions each based on the rarity of the reaction template that could have been used to recover the true outcome. Among the participants were chemistry and chemical engineering graduate students, postdocs, and professors. A performance comparison is shown in Fig. 4, illustrating that the model performs at the level of an expert chemist for this prediction task. Though we present the quantitative results of the benchmarking study, this is a small-scale qualitative comparison and should be treated as such. Details of this study can be found in the ESI.†

Because we are making predictions across a wide range of reaction types, performing well on this task necessitates an esoteric knowledge of chemical reactivity. This highlights a key reason why our model is a useful addition to the synthetic chemists toolbox. Machine learning models are well-suited to aggregate massive amounts of prior knowledge and apply them to new molecules, whereas most humans may only be able to recall the most common reaction types and general reactivity trends.

### Interpreting model understanding

In many deep learning applications, improved accuracy comes at the expense of interpretability. Some strategies for interpretation can be applied to models as black box mathematical functions,^36^ but the insight into model behavior is only indirect. Instead, the architecture of deep learning models should inherently enable some degree of interpretability as has been done in natural language processing.^37^

The global attention mechanism in the WLN, in addition to improving accuracy by accounting for reagent and other long-range effects, is designed to offer interpretability. The predicted reactivity of each atom pair is informed by the atoms' representations, which are in turn informed by their local and global environments. For example, a phenolic oxygen may not be inherently reactive, but in the presence of a strong base is amenable to various substitution or etherification reactions; in this scenario, we would expect oxygen to attend to a basic reagent in addition to its reacting partner. On the other hand, a diazo compound is of sufficient inherent reactivity that the local environment is all the model requires to predict the outcome accurately, and there is little information gained by attending to the global environment.

**Fig. 5:**
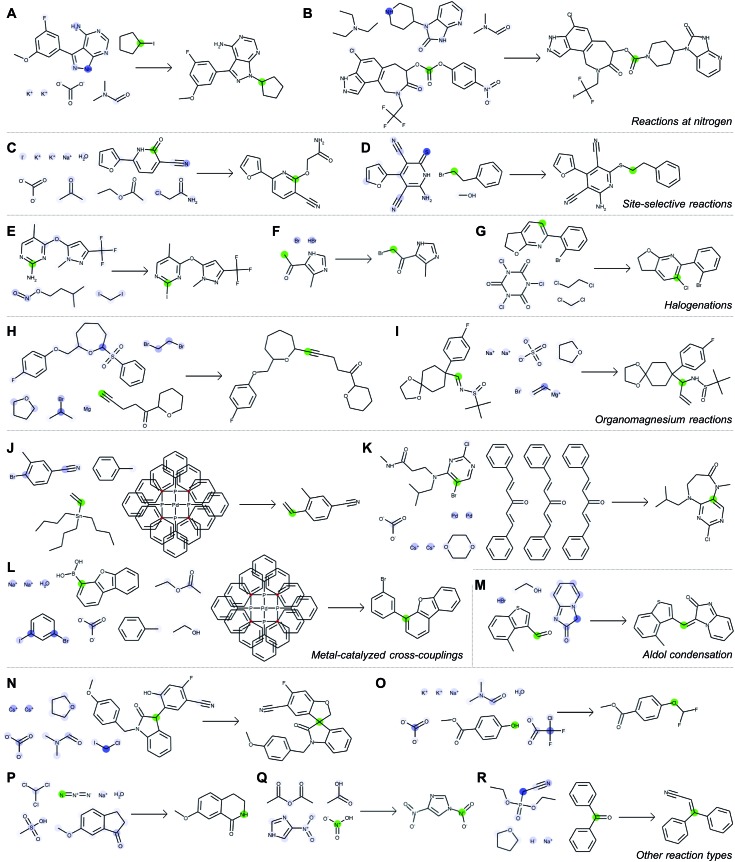
Correct predictions from the test set illustrating the neural model's learned chemical intuition and ability to make accurate predictions across a wide range of reaction families. For each example (A–R), we select one atom (green highlight) and examine to what extent every other atom contributed to the perceived reactivity at that location (blue highlight, where a darker color indicates stronger influence). Note that these model-generated structures do not follow traditional drawing standards (*e.g.*, use of abbreviations) in order to illustrate quantitative attention weights for each atom individually.

Fig. 5 depicts a series of correct predictions from the test set selected to showcase the diversity of correctly-predicted reaction types. The model is able to make accurate predictions for common alkylations (Fig. 5A and B), for cases of ambiguous regioselectivity (Fig. 5C and D), and for various methods of halogenation (Fig. 5E–G). It can distinguish between use cases of similar reagents (*e.g.*, alkyl magnesium species in Fig. 5H and I) and recognize common metal-catalyzed (Fig. 5J–L) or other C–C bond forming reactions (Fig. 5M). It also is able to predict complex preparations of quaternary carbon centers (Fig. 5N), specialized preparations of difluoromethyl ethers (Fig. 5O), Schmidt ring expansions (Fig. 5P), amine nitrosations (Fig. 5Q), and Wittig reactions (Fig. 5R).

With each reaction, we are able to examine which aspects of the reactant species most influences the model's perception of reactivity at the atom highlighted in green. A darker blue color indicates a larger attention score, which in turn indicates a stronger influence on its perceived reactivity. While these do not reveal information about reaction mechanisms directly, they are a significant step beyond a black box prediction of major products. The global attention mechanism reveals an explanation consistent with how we might justify the prediction in most cases by identifying suitable reaction partners and activating reagents.

For example, Fig. 5A indicates that the iodide-substituted carbon's reactivity is influenced by the presence of suitable reaction partners, namely the three most likely reaction sites on the purine; Fig. 5D is similar. For the Suzuki coupling in Fig. 5L, the aryl boronic acid carbon attends to both the iodo- and bromo-sites of its potential coupling partners in addition to sodium carbonate; however, as the reaction database often does not include catalysts even when employed in the actual experiments, the model does not rely on Pd(P(Ph)_3_)_4_ as an indication of reactivity. The tertiary carbon in Fig. 5N attends to chloroiodomethane most strongly, but also attends to Cs_2_CO_3_ as an activating base. In cases where reactivity can be correctly predicted based on solely local feature vectors (Fig. 5G and Q), attention scores are low and do not reveal additional insight.

Although only the correct rank-1 products are shown above, the model always predicts a probability distribution over multiple product species. An example of impurity prediction showing the top six predictions can be found in Fig. S1.† Several examples where the model predicts the recorded outcome as the second-most likely (rank-2) are shown in Fig. S2 and S3.† These “near-misses” are primarily cases where the model predicts an intermediate or a plausible side product, discussed in the ESI.† Fig. S4† shows additional examples where the recorded outcome is not predicted in the model's top-10 suggestions. The full set of predictions for all 40 000 test reaction examples is available online in addition to the original code and data.

## Conclusion

By designing a neural model to be aligned with how domain experts (chemists) might analyze a problem, we exceed state-of-the-art accuracy in reaction outcome prediction while simultaneously understanding how the model perceives chemical reactivity. Neural networks are therefore not resigned to be used as black box tools, nor are applications of machine learning techniques in chemistry restricted to off-the-shelf models. Model rationales provide insight to human experts and may support new human-machine collaborations for mechanism discovery. In predicting reaction outcomes, the model considers variables in a manner qualitatively similar to that of a human, namely the presence of suitable reaction partners and the effects of reagents and catalysts. The data set covers a broad range of common reaction types that would be found in medicinal and process chemistry settings. We believe that predictive models will have a large role to play in the future of automated experimentation, both to effectively use reaction data and to assist in the interpretation thereof.

## Funding

This work was supported by the DARPA Make-It program under contract ARO W911NF-16-2-0023 and the Machine Learning for Pharmaceutical Discovery and Synthesis Consortium; CWC received additional funding from the NSF GRFP under Grant No. 1122374.

## Conflicts of interest

Authors declare no competing interests.

## Supplementary Material

Supplementary information

Supplementary information

Supplementary information
