# Supplementary material for: A graph-convolutional neural network model for the prediction of chemical reactivity
Source: Chem Sci. 2018 Nov 26;10(2):370–7. doi: 10.1039/c8sc04228d (PMC6335848; doi:10.1039/c8sc04228d)
Supplement: Supplementary file 2 [file SC-010-C8SC04228D-s002.pdf]

Electronic Supplementary Material (ESI) for Chemical Science.
This journal is © The Royal Society of Chemistry 2018

Supporting Information

A graph-convolutional neural network model for the prediction of chemical reactivity

Connor W. Coley,[a] Wengong Jin,[b] Luke Rogers,[a] Timothy F. Jamison,[c] Tommi S. Jaakkola,[b] William H. Green,[a] Regina Barzilay,*[b] and Klavs F. Jensen*[a]

E-mail: regina@csail.mit.edu; kfjensen@mit.edu

[a] *Department of Chemical Engineering*
[b] *Computer Science and Artificial Intelligence Laboratory*
[c] *Department of Chemistry*

Massachusetts Institute of Technology
77 Massachusetts Avenue, Cambridge, MA 02139.

S1 Code and Data

All code used for model training can be found at `https://github.com/connorcoley/rexgen_direct`. The full data set of USPTO reactions used in this study can be found at the same link. We have included a “deployed” model that uses the trained weights of the model analyzed in detail in the manuscript.

S2 Methods

S2.1 Notation

Symbol	Meaning
u, v	atoms
$N(v)$	Set of atoms adjacent to v
$\tau(\cdot)$	ReLU activation function
$\sigma(\cdot)$	Sigmoid function
U, V, W	matrices in WLN/WLDN

S2.2 Weisfeiler-Lehman Network (WLN)

Weisfeiler-Lehman Network[32] is a type of graph convolutional network derived from Weisfeiler-Lehman (WL) graph kernel[38]. The architecture is designed to embed the computations inherent in WL graph kernel to learn isomorphism invariant representation of atoms. The atom representation is computed by iteratively augmenting the representation of adjacent atoms. Specifically, each atom v is initialized with a feature vector f_v indicating its atomic number, formal charge, degree of connectivity, explicit and implicit valence, and aromaticity. Each bond (u, v) is associated with a feature vector f_{uv} indicating its bond order and ring status. In each iteration, we updated atom representations as follows:

$$f_v^l = \tau\left(U_1 f_v^{l-1} + U_2 \sum_{u \in N(v)} \tau(V_1 f_u^{l-1} + V_2 f_{uv})\right) \quad (1 \leq l \leq L)$$

where f_v^l is the atom representation at the lth iteration, initialized with $f_v^0 = f_v$ atom features. U_1, U_2, V_1, V_2 are model parameters to be learned, shared across all L iterations. The final local atom representations are computed as

$$c_v = \sum_{u \in N(v)} W_1 f_u^L \odot W_2 f_{uv} \odot W_3 f_v^L$$

We refer the reader to 32 for more details about the mathematical intuition and justification of the WLN.

S2.3 Attention Mechanism

The atom embedding c_v only record local chemical environment, namely atoms and bonds accessible within L steps from atom v. Even if L were very large, c_v could not encode any information about other reactant molecules, as information cannot be propagated between two reactant molecules that are disconnected. We argue that it is important to enable information to flow between distant or disconnected atoms. For example, the reaction center may be influenced by certain reagents that are disconnected from reactant molecules. In this case, it is necessary for atom representation c_v to encode such distal chemical effects. Therefore, we propose to enhance the model in previous section with an attention mechanism[39].

Specifically, let α_{vz} be the attention score of atom v upon atom z. The "global" atom representation $\tilde{c}_v$ of atom v is calculated as the weighted sum of all reactant atoms where the weight comes from the attention module:

$$\alpha_{vz} = \sigma(u^T \tau(P_a c_v + P_a c_z + P_b b_{vz}))$$

$$\tilde{c}_v = \sum_v \alpha_{vz} c_z$$

The attention score is computed based on "local" atom representations c_v from WLN.

S2.4 Reaction Center Prediction

The WLN is trained to predict reaction center, a set of changes in graph connectivity that describe the difference between reactant molecules and major products. Mathematically, a reaction center is a set $\{(u, v, b)\}$, where (u, v) is a pair of atoms whose connecting bond has changed to type b. We predict the likelihood of (u, v, b) being in reaction center by passing atom representations from WLN through another neural network:

$$s_{u,v,b} = \sigma\left(u_b^T \tau(M_a \tilde{c}_u + M_a \tilde{c}_v + P_a c_u + P_a c_v + M_b f_{uv})\right)$$

The above neural network is jointly optimized with WLN to minimize the cross entropy loss:

$$-\sum_{u,v,b;u\neq v} y_{u,v,b}\log s_{u,v,b} + (1-y_{u,v,b})\log(1-s_{u,v,b})$$

where $y_{u,v,b}=1$ iff (u,v,b) is in the reaction center, and the above loss sweeps over every pair of atoms and bond types (including no bond).

S2.5 Candidate Ranking via Weisfeiler-Lehman Difference Network (WLDN)

At the stage of candidate reaction evaluation, we have a list of candidate products $\{p_0, p_1, \cdots, p_m\}$ given a set of reactant molecules r. The goal is to learn a scoring function that ranks the true product p_0 to be the highest. The challenge in ranking candidate products is again representational. We must learn to represent (r,p) in a manner that can focus on the key difference between the reactants r and products p, while also incorporating the necessary chemical contexts surrounding the changes.

The architecture of WLDN is designed to highlight such differences. Specifically, it has two components. The first component is a Siamese WLN that learns atom representation of reactant r and candidate products p_i. Let $c_v^{(p_i)}$ be the learned atom representation of atom v in candidate product molecule p_i. We define difference vector $d_v^{(p_i)}$ pertaining to atom v as follows:

$$d_v^{(p_i)} = c_v^{(p_i)} - c_v^{(r)}$$

Because the reactants and products are atom-mapped, we can use v to refer to the same atom in different molecules. The second component of WLDN is another WLN that operates on the difference graph between reactants and products. A difference graph $D(r,p_i)$ is defined as a molecular graph which has the graph structure as p_i, with atom v's feature vector replaced by $d_v^{(p_i)}$. Operating on the difference graph has several benefits. First, in $D(r,p_i)$, atom v's feature vector deviates from zero only if it is close to the reaction center, thus focusing the processing on the reaction center and its immediate context. Second, $D(r,p_i)$ explicates neighbor dependencies between difference vectors. The WLDN maps this graph-based representation into a fixed-length vector, by applying the second WLN on top of $D(r,p_i)$:

$$h_v^{(p_i,l)} = \tau\left(U_1 h_v^{(p_i,l-1)} + U_2 \sum_{u\in N(v)} \tau\left(V_1 h_u^{(p_i,l-1)} + V_2 f_{uv}\right)\right) \qquad (1\leq l\leq L)$$

$$g_v^{(p_i)} = \sum_{u\in N(v)} W_1 h_u^{(p_i,L)} \odot W_2 f_{uv} \odot W_3 h_v^{(p_i,L)}$$

where $h_v^{(p_i,0)} = d_v^{(p_i)}$. Note that though with the same notation, matrices U_*, V_*, W_* are distinct parameters from the WLN used in the reaction center prediction. We use the same character for notational convenience.

Let $RC(p_i) = \{(u_i, v_i, b_i)\}$, the set of bonds that changed from the reactant r to product p_i. The final score of candidate p_i is:

$$s(p_i) = u^T \tau\left(M \sum_{v\in p_i} g_v^{(p_i)}\right) + \sum_{(u,v,b)\in RC(p_i)} s_{u,v,b}$$

Compared to 29, we augment the WLDN with the quantitative scores $s_{u,v,b}$ for each bond change in reaction center prediction. This is beneficial as the candidate outcomes produced by combinations of more likely bond changes are themselves more likely to be the true outcome.

S3 Additional Results

S3.1 Number of bond changes per reaction

The combinatorics of the enumeration scales poorly with the number of bond changes allowed per reaction. As stated in the main text, the number of candidates per reaction is bounded by

$$\sum_{n=1}^{5} \binom{K}{n}$$

Where we have allowed up to 5 simultaneous bond changes and $K = 16$ (i.e., select up to 5 bond changes from the 16 most likely bond changes) in our later evaluations. The choice of 5 was motivated by an analysis of the number of bond changes in training and validation examples shown in Table S1. We sacrifice 0.1-0.2% loss in maximum possible predictive accuracy through this limitation, but significantly restrict the number of candidates that must be ranked. To allow predictions of the remaining 0.1-0.2%, it would be possible to have a dynamic upper limit of the number of simultaneous bond changes that takes into account what those bond changes are (e.g., to allow complex pericyclic reactions that may have many bond rearrangements).

Table S1 Number of simultaneous bond changes for reaction examples in the USPTO dataset used in this study. Very few reactions involve 6 bond changes, so the candidate enumeration is limited to selecting only up to 5.

	Number of simultaneous bond changes					
Dataset	1 [%]	2 [%]	3 [%]	4 [%]	5 [%]	6 [%]
Training	17.1	55.1	19.5	6.6	1.4	0.2
Validation	17.4	54.9	19.7	6.5	1.4	0.1
Testing	17.4	55.0	19.8	6.4	1.3	0.1

S3.2 Computational cost of training and prediction

An important aspect of predictive model performance is speed or computational cost, particularly in cases where the model may be applied in a high throughput virtual screen. All experiments were run using a single NVIDIA Titan X graphics card. Training of the Reaction Center Prediction model (WLN) completed after 19 hours (140,000 minibatches of 20, an average of 24 ms per example). Training of the Candidate Ranking model (WLDN) completed after 72 hours (2,400,000 minibatches of a single reaction and its candidate outcomes, an average of 108 ms per example).

Prediction times for the 40,000 test examples were 28.5 minutes and 141 minutes respectively using a single Titan X GPU and a single data preprocessing thread. This translates to a throughput of 43 ms/example and 212 ms/example, respectively. It's important to note that when making these predictions, preprocessing occurs on a single thread (i.e., converting a SMILES to the reactant graph, combinatorically enumerating candidate outcomes and determining their validity). The throughput for testing âĂŞ as implemented âĂŞ is currently limited by these CPU-related tasks, particularly for the candidate ranking model. These preprocessing steps could be trivially parallelized at the reaction level as was done during training and would enable inference times below that of training times (i.e., below 24 and 108 ms per example for each model).

S3.3 Reaction prediction performance compared to Schwaller et al.'s single product subset

In 28, Schwaller et al. report their model performance on single product reactions in addition to those in the full data set. Only 3.4% of test examples in the Jin et al. data set have multiple species in the products (e.g., counterions, amine salts). However, their neural translation model is currently not designed to predict multiple species separated by a "." SMILES token. Table S2 shows the comparison using the subset of 38,648 single product examples. While the difference in performance is less significant than with the full test set, our graph-based model still achieves several percent higher accuracy.

Table S2 Performance in reaction prediction when only testing on the 38,648/40,000 reactions with a single reported product (i.e., no counterion or salt).

Method	Top-1 [%]	Top-2 [%]	Top-3 [%]	Top-5 [%]
Sequence-to-sequence (28)	83.2	87.7	89.2	-
This work	**86.4**	**91.3**	**92.9**	**94.2**

S3.4 Reaction prediction performance compared to Bradshaw et al.'s "linear mechanism" subset

In 26, Bradshaw et al. formulate the task of forward prediction as a predicting a sequence of electron paths as a pseudo-mechanism. However, of the ca. 470k USPTO reactions representing the combined training, validation, and test set used in this study and used by Jin et al. and Schwaller et al. previously, only 73% of examples can be represented in this manner. That is, 27% of reactions from

this data set are impossible to predict because they do not fit within this linear framework. The first five test reactions that are not in their subset are a reductive amination, a total deprotection of a tertiary amine to a primary amine, a thioether oxidation to a sulfoxide, thiourea addition to an alkyl iodide, and an alkene ozonolysis to an aldehyde. These are reaction types that one should expect these models to be able to predict. Our formulation of reactions as sets of bond order changes allows 98.6% of test examples to be represented and reconstructed (note: we consider the remaining 1.4% as failed predictions in all evaluations).

In their preprint, Bradshaw et al. show a comparison between ELECTRO and the previous models of Jin et al. and Schwaller et al. However, a very important point must be made: the focus on reactions that can be described as sequential electron movements represents a significant restriction on possible outcomes. This added problem structure simplifies the prediction task and, in the context of our model, would restrict the number of valid enumerated candidates. In general, one should expect it to be easier to perform well on a narrower task with a model that is tailored to that task's scope.

We make a comparison using the test subset of 29,360 reactions provided by Bradshaw et al.. For the sake of this evaluation, because the exact training data is not available, we use the trained models designed for the broader prediction task over the whole data set. While it would be possible to restrict candidate enumeration to only include products consistent with the restricted reaction scope, we have not done so in this comparison. The results are shown in Table S3. Although the model described in this study is designed for a more general prediction task, it still outperforms the ELECTRO model in top-1 and top-2 accuracy by a small margin.

Table S3 Performance in reaction prediction when only testing on the 29,360/40,000 reactions able to be predicted by the ELECTRO model; our model was not designed to take advantage of the narrower prediction scope but still achieves slightly higher top-1 and top-2 accuracies.

Method	Top-1 [%]	Top-2 [%]	Top-3 [%]	Top-5 [%]
ELECTRO (26)	87.0	92.6	**94.5**	**95.9**
This work	**88.3**	**92.9**	94.2	95.3

S3.5 Human benchmarking

Supporting information file "Human benchmarking (80 examples) and answers" contains all 80 questions from the human benchmarking test and their answers. Recorded reactants and products are shown in black. For reactions where the model did not predict the true (recorded) product as its top prediction, the predicted outcome is shown in red. Reactions from the test set of 40,000 reactions were divided into 8 categories based on the rarity of the retrosynthetic reaction template required to reproduce the example using a template-based method. Ten reactions were randomly selected from each of these eight categories to cover both common and rare reactions. Human performers were given depictions of the reactant molecules and asked to draw or otherwise indicate the expected major product, which exactly matches the model's prediction task; no explicit time limit was provided. To evaluate the model on the same data set of 80 test reactions, the model was *not* given explicit information about which species are known a priori to be reagents, to make it a fair comparison.

The comparison between the model and human performers serves to indicate that this is a nontrivial prediction task and that the model is able to perform at the level of an expert human. A more tightly controlled, larger-scale study would be required for a more rigorous comparison.

S3.6 Application to impurity prediction

S3.7 Near-miss predictions

Fourteen mispredictions are shown in Fig. S2 and Fig. S3 where the model predicts the recorded outcome with rank 2; the rank 1 prediction is shown in red. In all cases, the model makes reasonable predictions given the problem formulation and information it has access to. Fig. S2A shows an example of regioisomerism, where the model predicts an isopropylation at the 1 position of the indazole, but the recorded product is at the 2 position. Fig. S2B is an examply where the recorded reaction is neutralization of a deprotonated carboxylic acid; under our formalization of reactions as changes in bond order, this would be seen as âĂIJno reactionâĂİ âĂŞ an outcome that the model is not allowed to predict. Given that the model must make a prediction of a product with modified bond orders, it defaults to a fairly common reaction from the database, a deprotection, although Cbz is generally stable to base. Fig. S2C is another case of regioisomerism, where condensation can occur on either side of the methyl butyl ketone. Fig. S2D is a case of complex regioselectivity, where the model identifies the recorded iodination as likely, but believes a different site to be more so. One would expect that both products would be observed experimentally. The model prediction for Fig. S2E can be seen as the single-step intermediate prior to a second amidation to the recorded outcome; likewise, the model prediction for Fig. S2F is an intermediate prior to elimination to form the recorded product. Fig. S2G is a C-N bond forming reaction where the rank-1 and rank-2 predictions correspond to the two most likely sites of addition; the distinction between the two is subtle, resulting from a distant bromine that breaks the molecule's symmetry. The site that the model predicts as more likely does not match what was recorded.

Fig. S3A shows the acylation of an alkene mispredicted as the esterification of an enol. Fig. S3B is a case of regioisomerism similar to Fig. S2G, where the asymmetry between the two reactive sites is subtle. The model rank-1 prediction for Fig. S3C is singly-alkylated

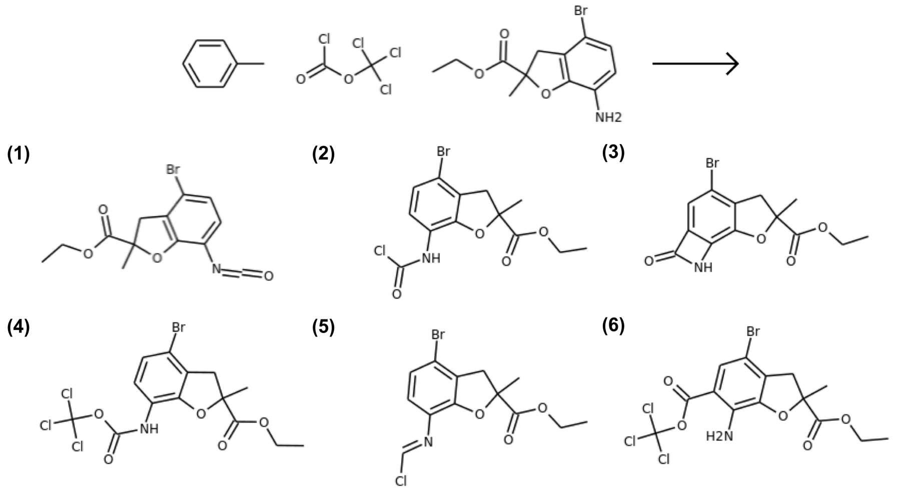

Fig. S1 Example of impurity prediction for the preparation of an isocyanate. The model correctly predicts the recorded product (rank 1, >99% confidence), but also suggests several minor side products (only ranks 2-6 shown) as potential outcomes.

intermediate to the recorded and rank-2 prediction, the double-alkylated ethanolamine. The presence of both HF and pyridine for the epoxide opening in Fig. S3D leads to the prediction of both regioisomers, whereas only one is recorded. The rank-1 and rank-2 predictions of Fig. S3E are tautomers, highlighting the fact that while the SMILES strings of each species are sanitized and canonicalized by RDKit, there are additional standardization steps that might reveal some products to be equivalent. The recorded reaction of Fig. S3F suggests that the example may be missing a reagent; the model, required to make a prediction and in the absence of any better candidates, suggests an unlikely SNAr between chloropyridine and triethylamine. Indeed, the true reaction example includes potassium trifluoro(vinyl)borate (PFIZER LIMITED - US2007/197478, 2007, A1). The recorded Chan-Lam coupling in Fig. S3G is mispredicted as a Suzuki coupling. Despite the absence of any Pd catalyst, the model has learned that this is a very likely outcome and scores it higher than the true product.

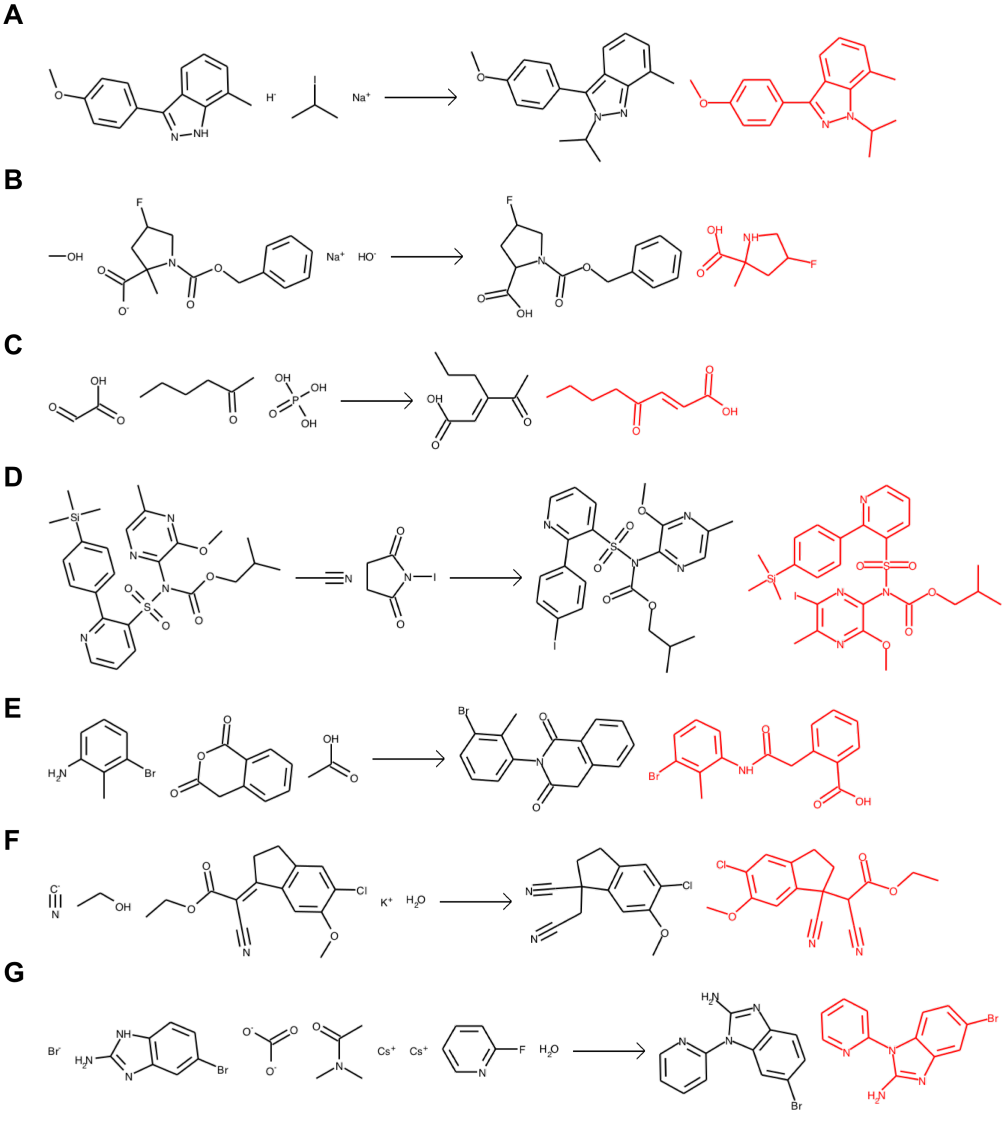

Fig. S2 Miscellaneous “near-miss” mispredictions from the test set where the model proposes the recorded outcome as its rank-2 prediction (black); the incorrect rank-1 prediction is shown in red. (A) mispredicted regioselectivity for indazole N-alkylation; (B) ester hydrolysis predicted, acid neutralization recorded; (C) kinetically-favored aldol condensation predicted, thermodynamically-favored aldol condensation recorded; (D) misprediction of iodination site selectivity, (E) ring-opening amidation predicted, double-amidation recorded; (F) misprediction of ester elimination upon -ene cyanation; (G) misprediction of N-alkylation selectivity.

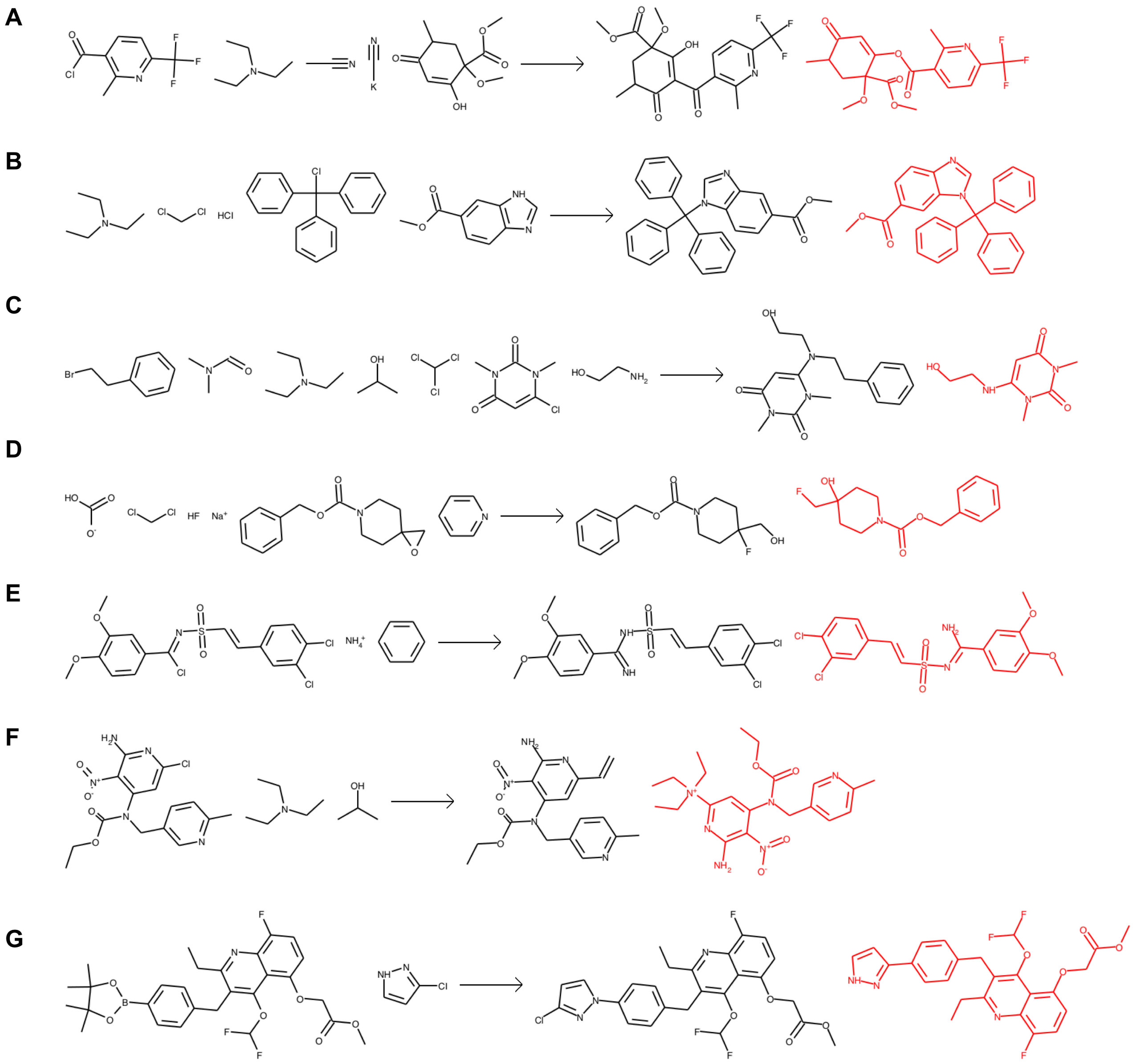

Fig. S3 Miscellaneous "near-miss" mispredictions from the test set where the model proposes the recorded outcome as its rank-2 prediction (black); the incorrect rank-1 prediction is shown in red. (A) esterification predicted, alpha carbon acylation recorded; (B) mispredicted regioselectivity of N-alkylation (C) single N-alkylation predicted, double N-alkylation recorded; (D) misprediction of epoxide opening selectivity; (E) prediction of tautomer; (F) SNAr predicted, alkenation recorded; (G) Suzuki coupling predicted, N-alkylation recorded.

S3.8 Complete-miss predictions

Nine mispredictions are shown in Fig. S4 where the model does not predict the recorded outcome in its top ten predictions; the rank 1 prediction is shown in red. In most cases, the model makes reasonable predictions given the problem formulation and information it has access to. Fig. S4A shows a two-step azidation followed by a reduction with sodium borohydride, where the model only predicts the azide product and does not continue to the aniline. Fig. S4B shows a chlorination reaction where the recorded outcome is a substitution at the aryl nitro group; the model instead predicts that the phallic anhydride will open to form the acid chloride. Fig. S4C is a case where the recorded outcome does not make physical sense, due to the presence of an additional carbon atom unlikely to be contributed by any of the reagents. Fig. S4D is similar, where the n-propylation is challenging to explain based on the recorded reactant/reagent species. In Fig. S4E, it is unclear what the source of the carbonyl carbon and oxygen are in the recorded outcome species, but the predicted outcome is quite reasonable. The recorded and predicted outcomes in Fig. S4F are tautomers, yet this is considered a misprediction due to having distinct SMILES representations. The misprediction in Fig. S4G is a legitimate one, where the model perhaps doesn't understand the role of mercury oxide and falls back on predicting an alkyne reduction. The spiroether motif is not terribly common in the patent set, so it is likely that the model has not seen enough of these examples to confidently predict this type of reaction. The recorded outcome in Fig. S4H is essentially âĂIJno reactionâĂİ, and the model's prediction of the acid chloride makes more physical sense. The final example shown in Fig. S4I is what appears to be a simple N-alkylation with an alkyl bromide, but the recorded product is the imine, rather than the amine.

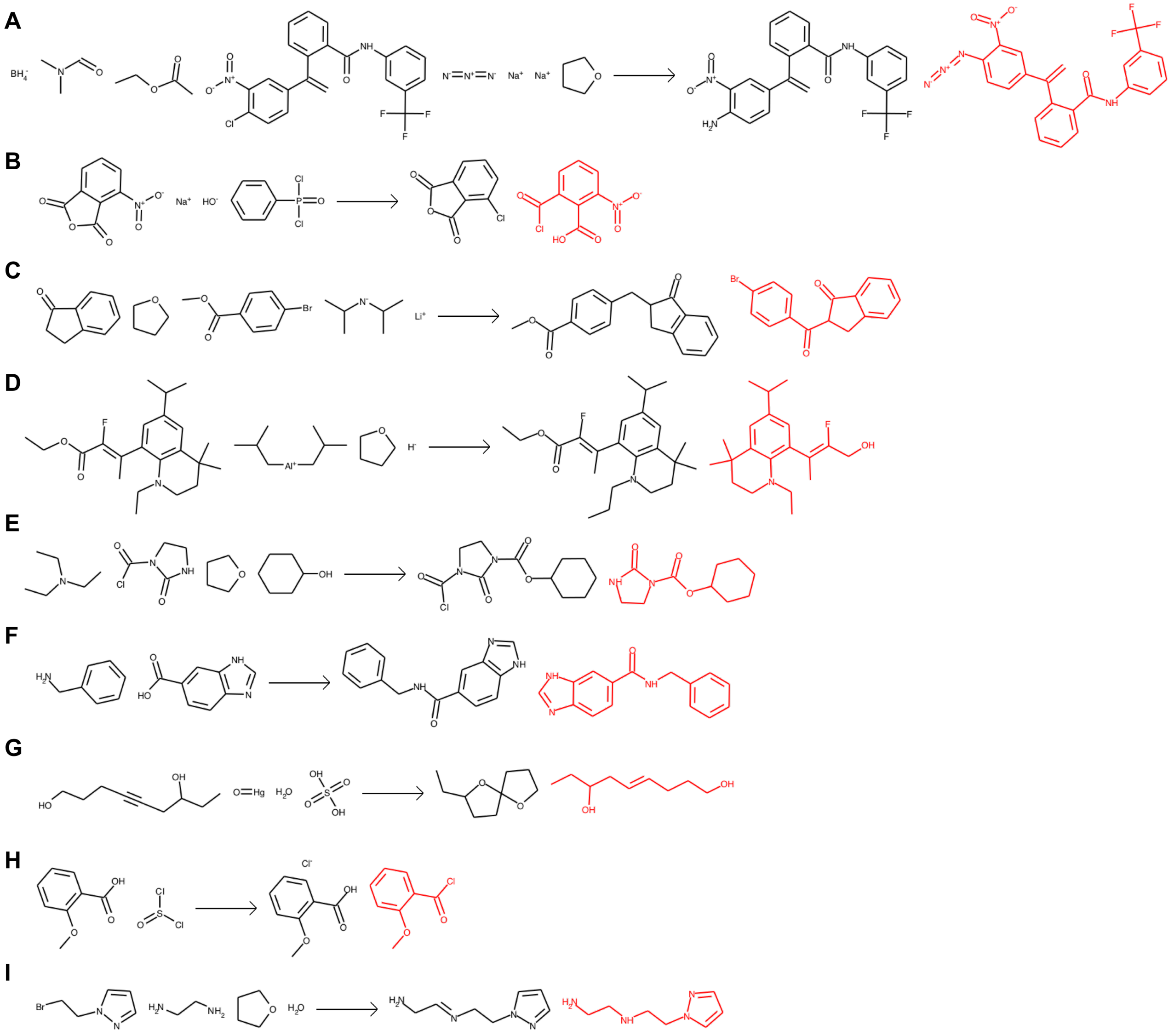

Fig. S4 Miscellaneous "complete-miss" mispredictions from the test set where the model does not propose the recorded outcome (black) in any of its top-10 predictions; the incorrect rank-1 prediction is shown in red. (A) azidation predicted, two-step azidation and reduction recorded; (B) ring-opening chlorination of anhydride predicted, nitro substitution recorded; (C) alpha acylation predicted, unexplainable alpha arylation recorded with additional carbon; (D) ester cleavage predicted, unexplainable n-propylation recorded; (E) esterification predicted, unexplainably amidation recorded with additional carbonyl; (F) amidation predicted, equivalent amidation tautomer recorded; (G) alkyne reduction predicted, spiroether formation recorded; (H) chlorination of carboxylic acid predicted, disassociated chloride salt recorded; (I) N-alkylation predicted, unexplainable N-alkylation to imine recorded.

S4 Data set analysis

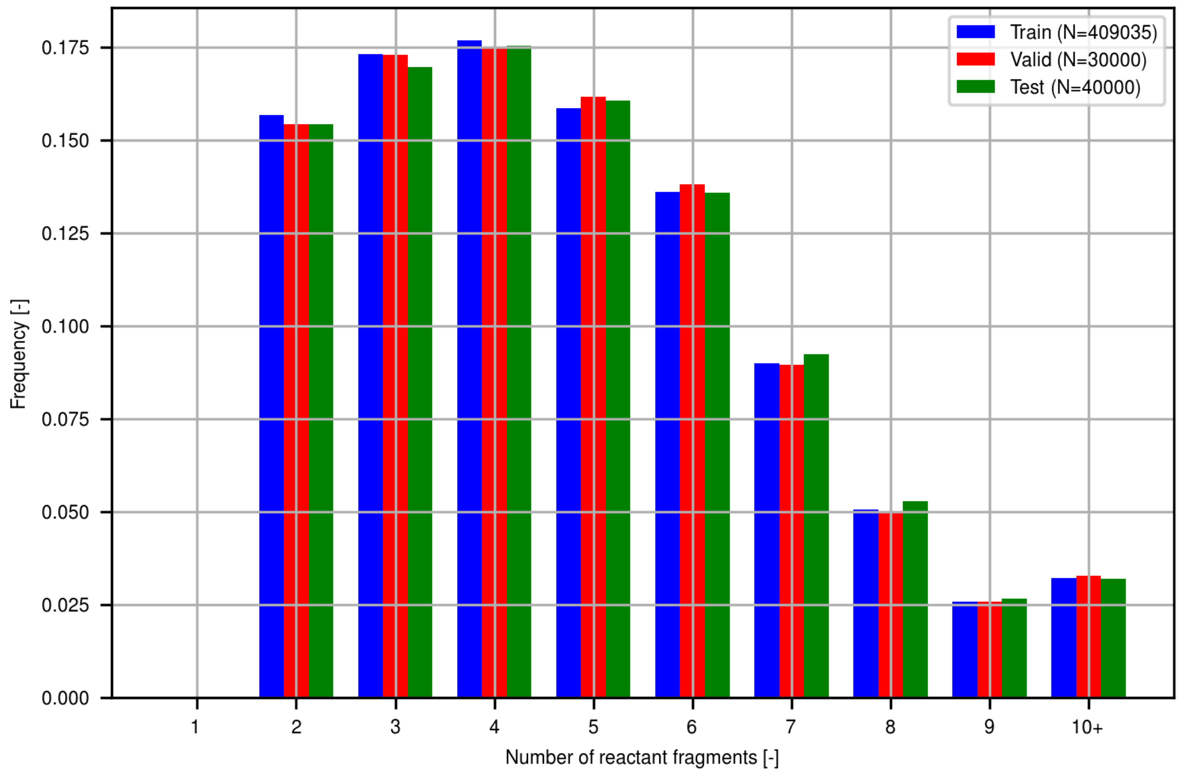

Fig. S5 Analysis of the USPTO data set used in this study in terms of the number of reactant fragments present in each reaction SMILES as determined by the presence of the period (".") symbol.

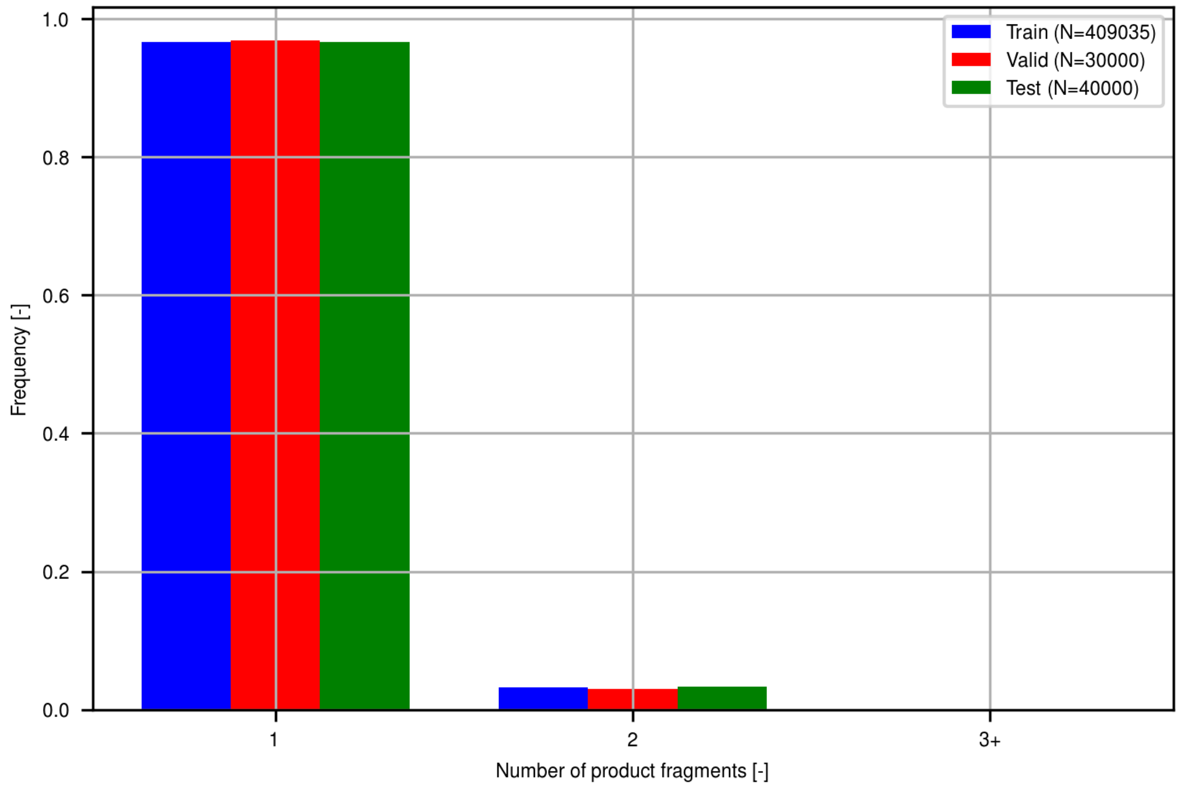

Fig. S6 Analysis of the USPTO data set used in this study in terms of the number of product fragments present in each reaction SMILES as determined by the presence of the period (".") symbol. The relatively few examples with multiple product species are primarily salts (e.g., amine HCl salts) or counterions.

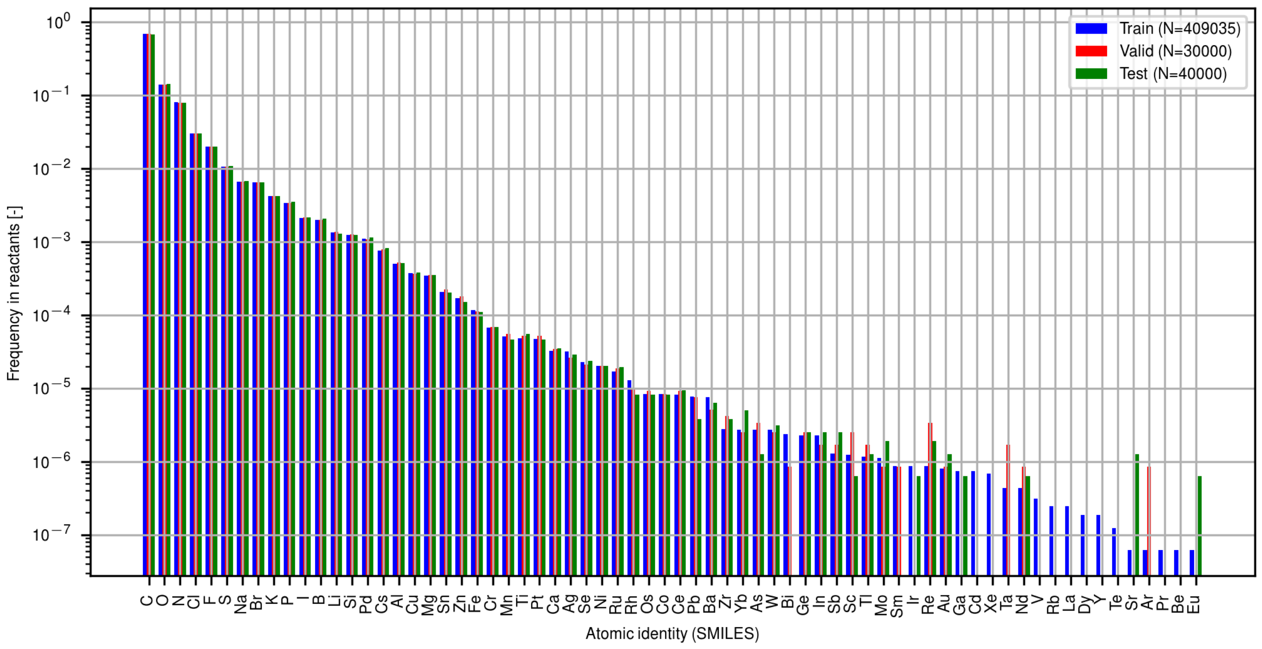

Fig. S7 Analysis of the USPTO data set used in this study in terms of the diversity of atomic identities appearing in the reactant species. Note the log scale of the y axis.

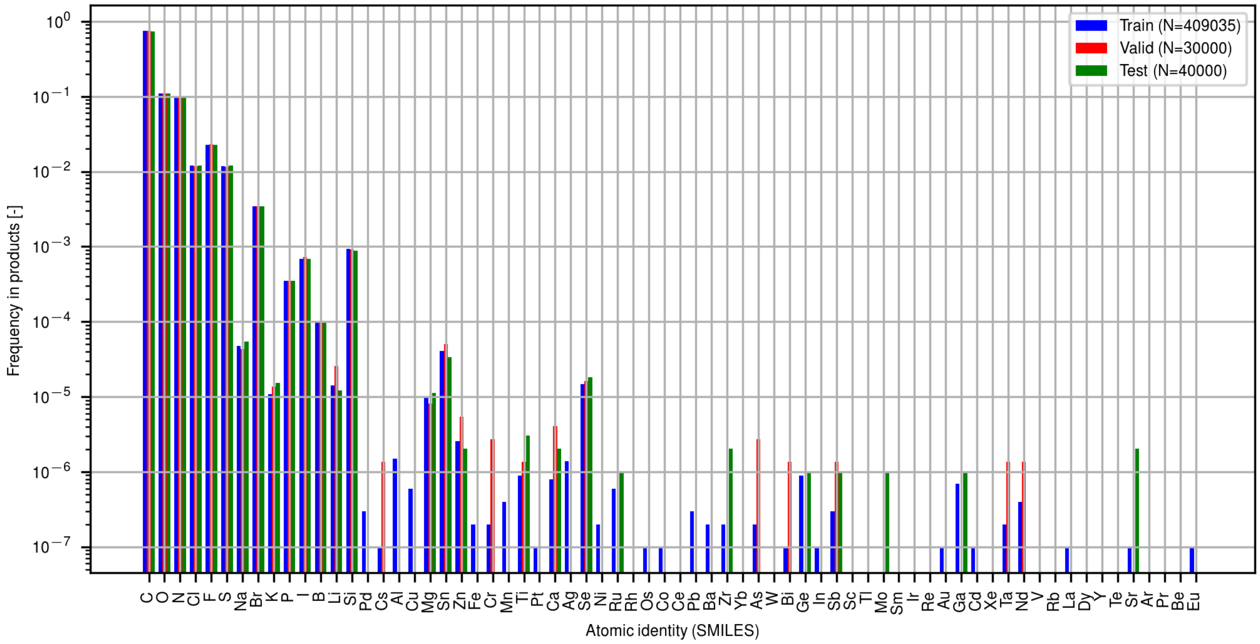

Fig. S8 Analysis of the USPTO data set used in this study in terms of the diversity of atomic identities appearing in the product species. Note the log scale of the y axis. Comparison to Fig. S7 shows that there are fewer distinct heavy atoms that appear in products as compared to reactants; many predominantly appear in reagents or catalysts.

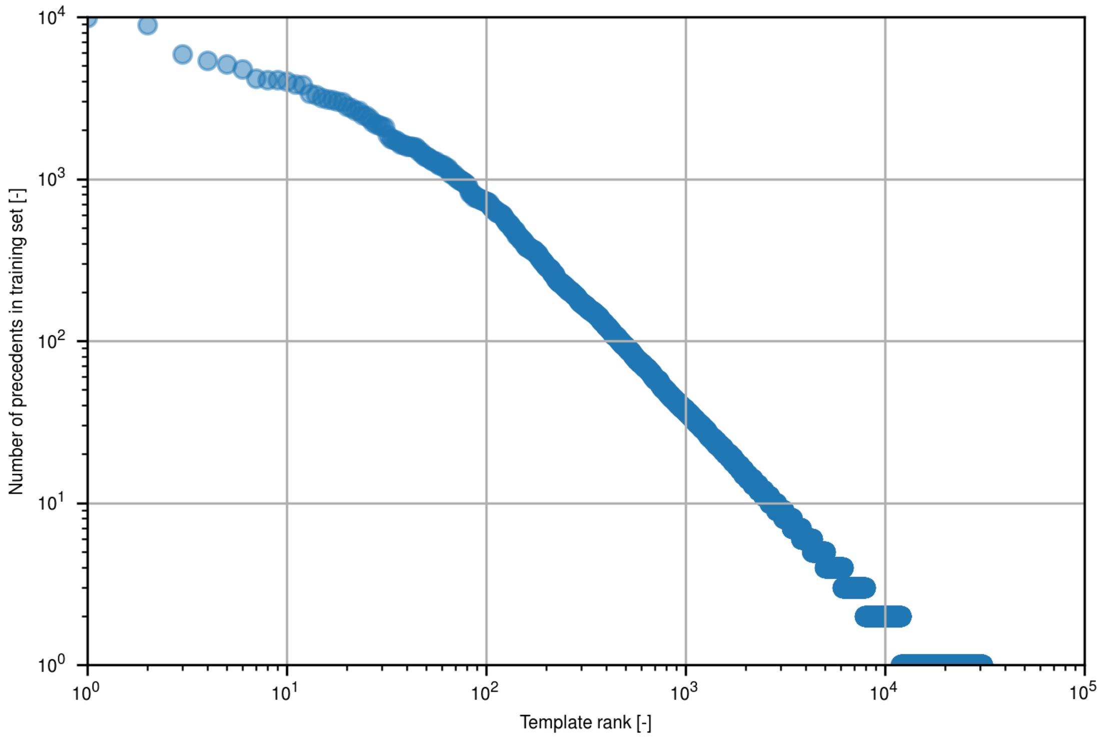

Fig. S9 Popularity of reaction templates extracted from the training data set of ca. 410k reactions showing an inverse power law relationship. Of the 30,762 distinct reaction templates, 18,725 have a *single* precedent reaction and 25,756 have fewer than five.
