# Supplementary material for: A graph-convolutional neural network model for the prediction of chemical reactivity
Source: Chem Sci. 2018 Nov 26;10(2):370–7. doi: 10.1039/c8sc04228d (PMC6335848; doi:10.1039/c8sc04228d)

Electronic Supplementary Material (ESI) for Chemical Science.
This journal is © The Royal Society of Chemistry 2018

1.

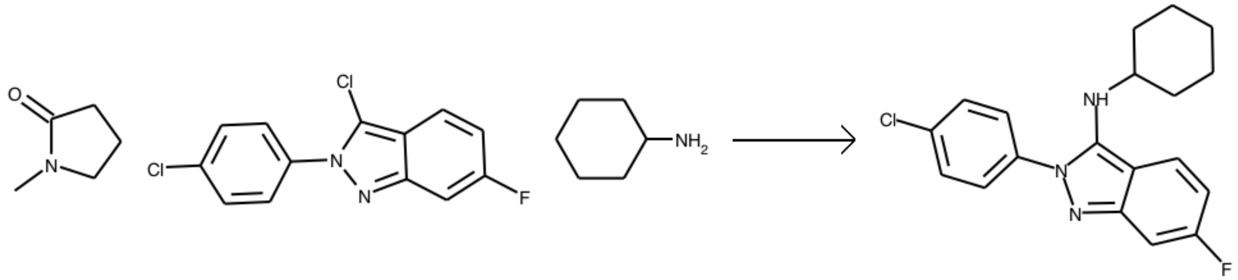

2.

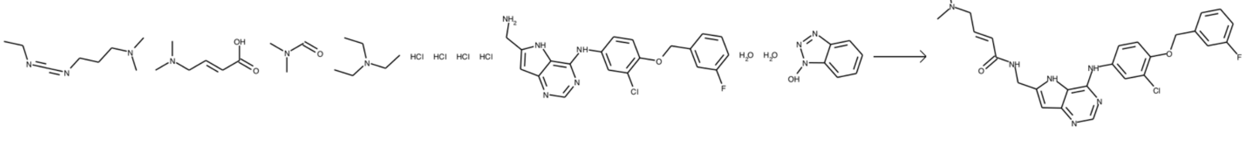

3.

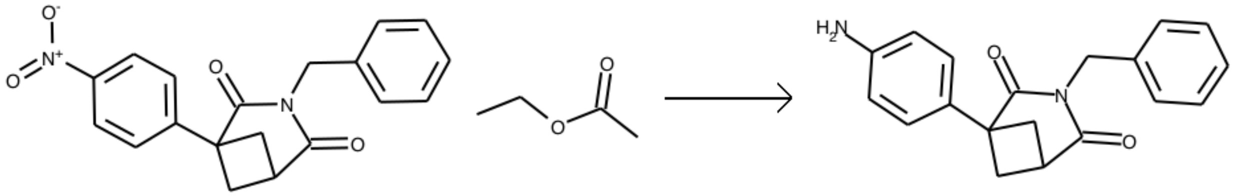

4.

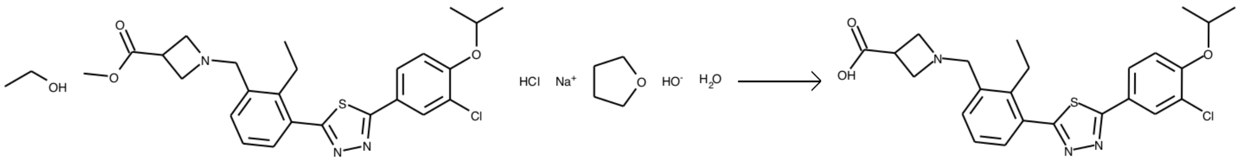

5.

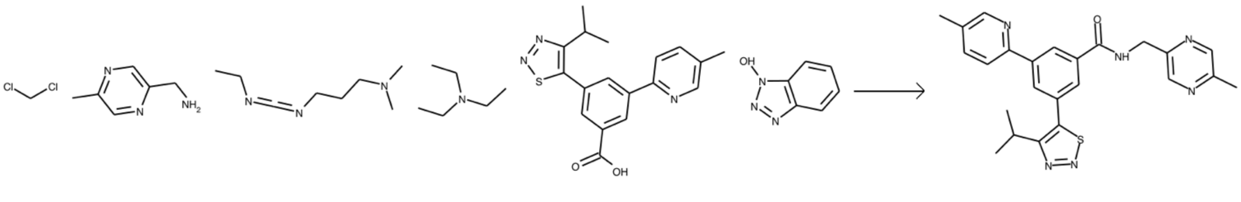

6.

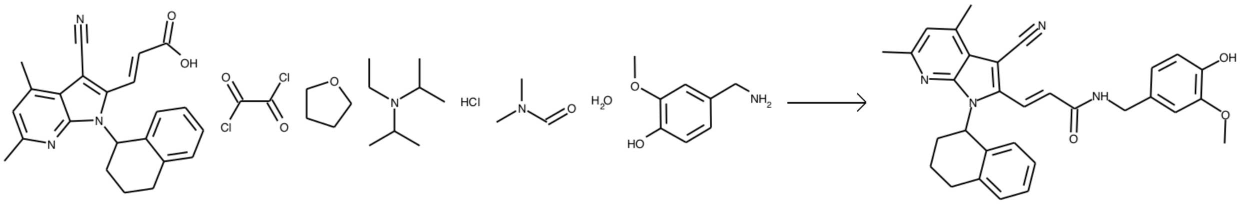

7.

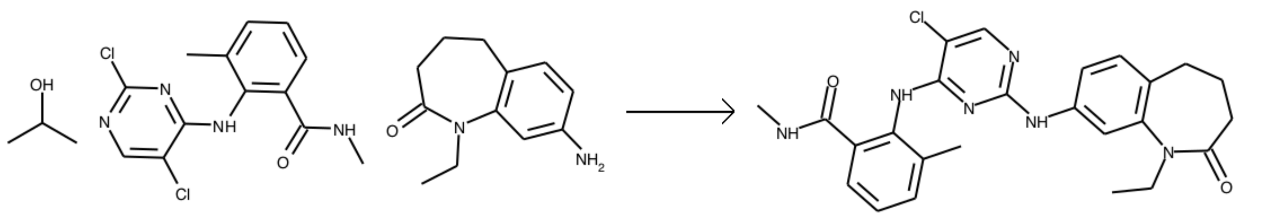

8.

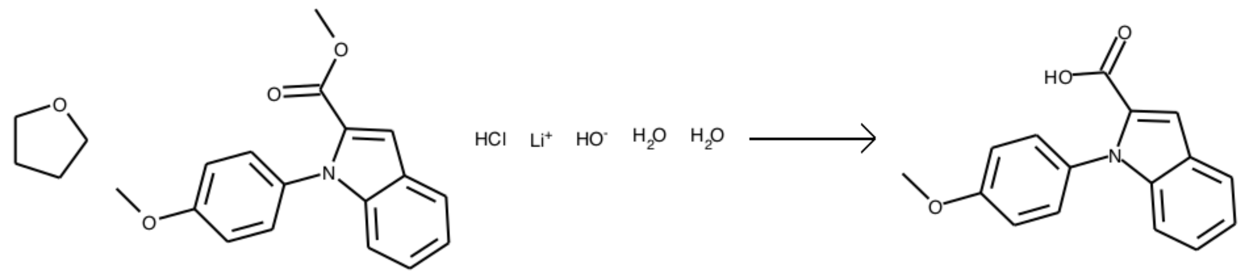

9.

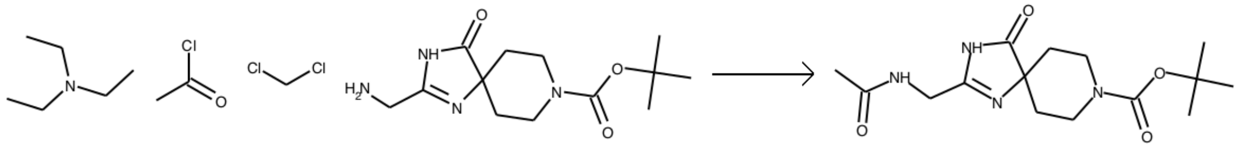

10.

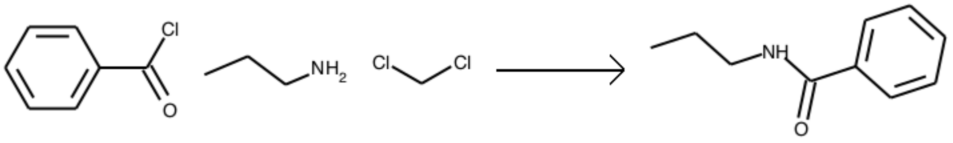

11.

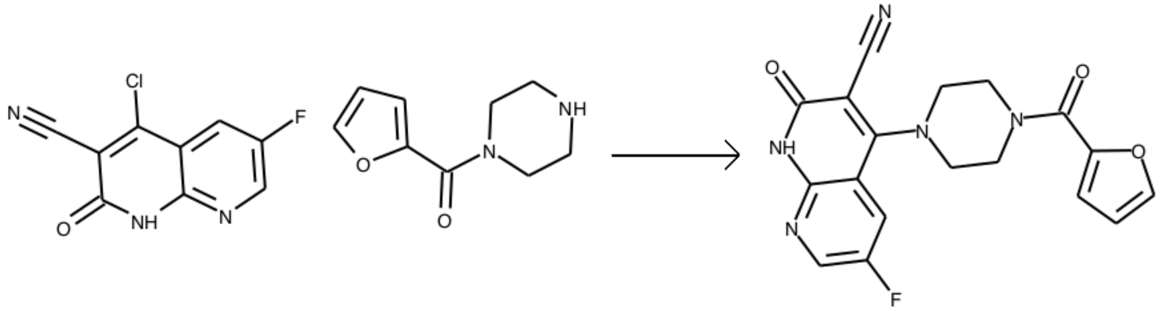

12.

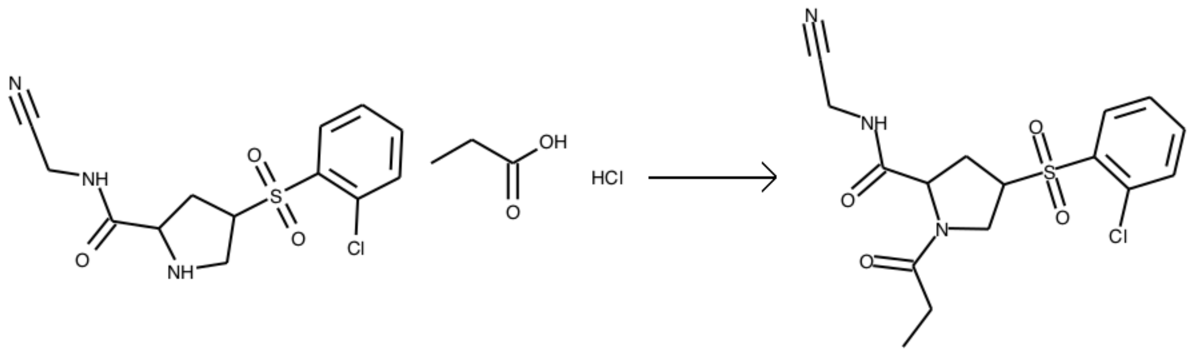

13.

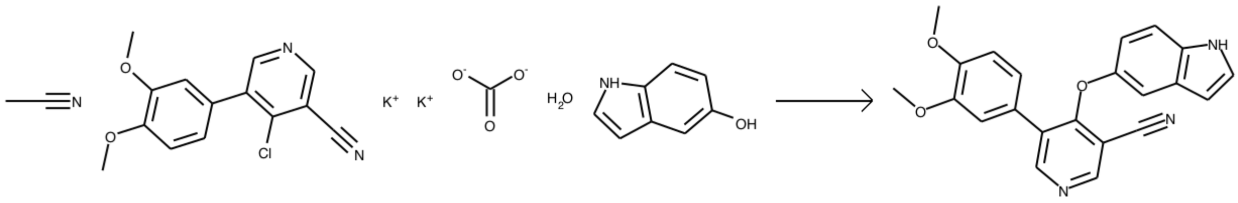

14.

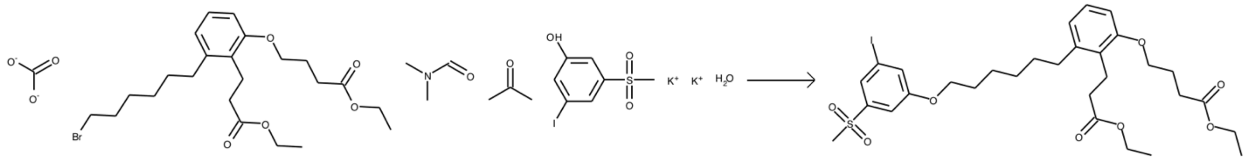

15.

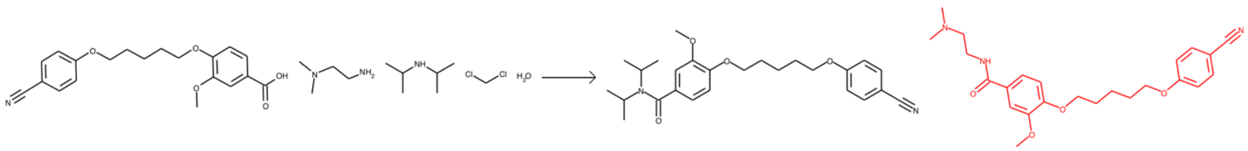

16.

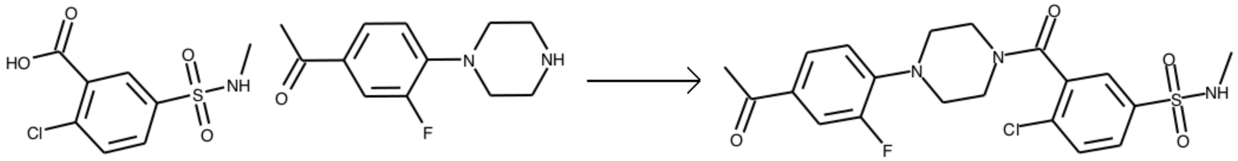

17.

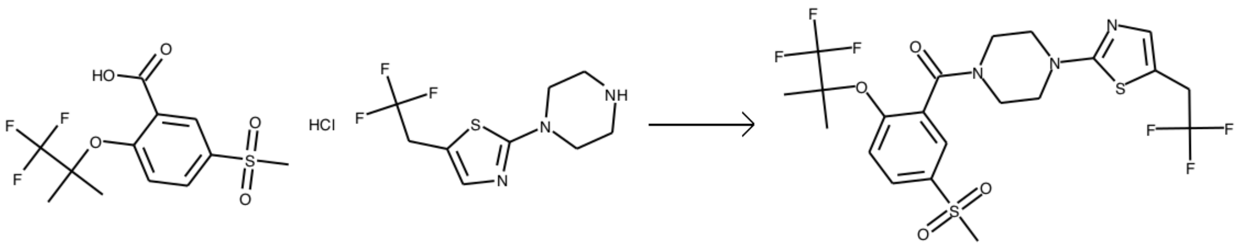

18.

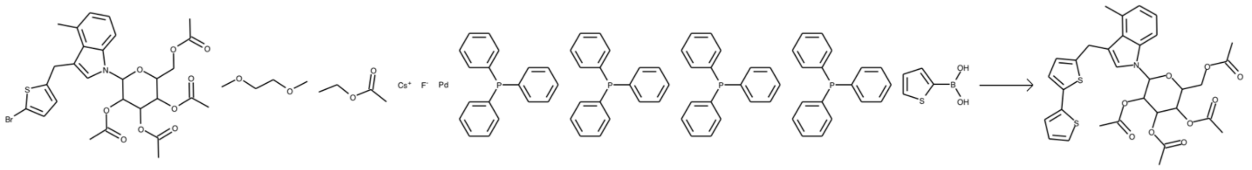

19.

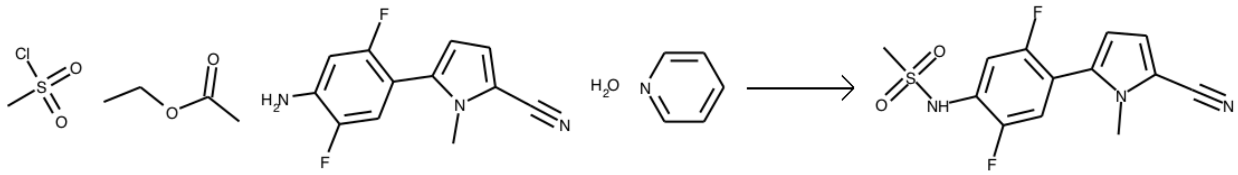

20.

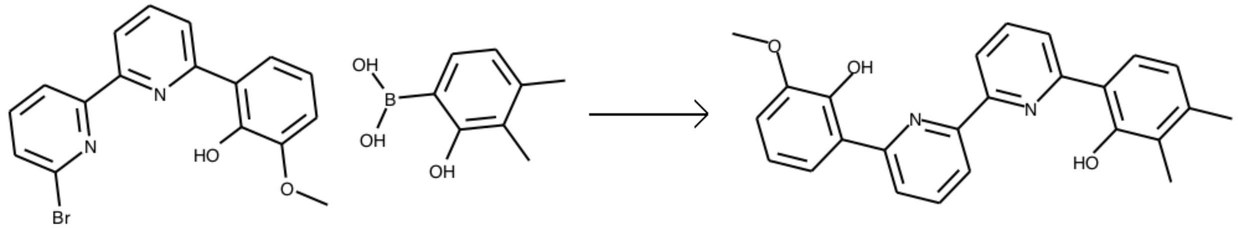

21.

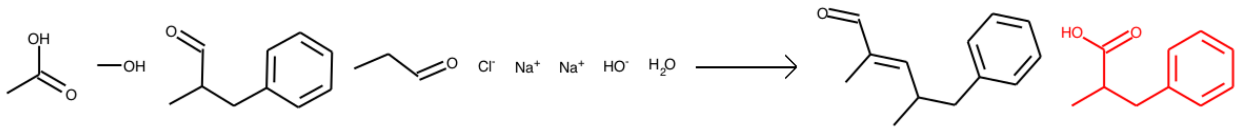

22.

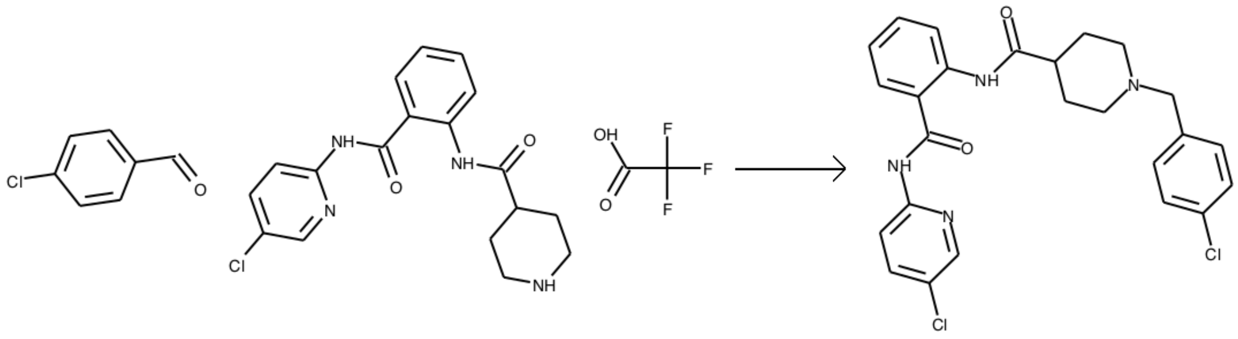

23.

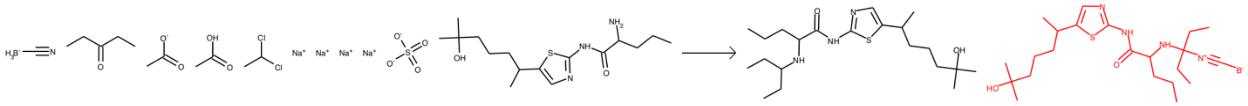

24.

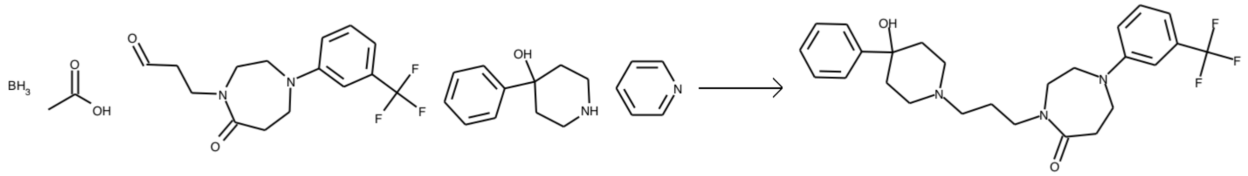

25.

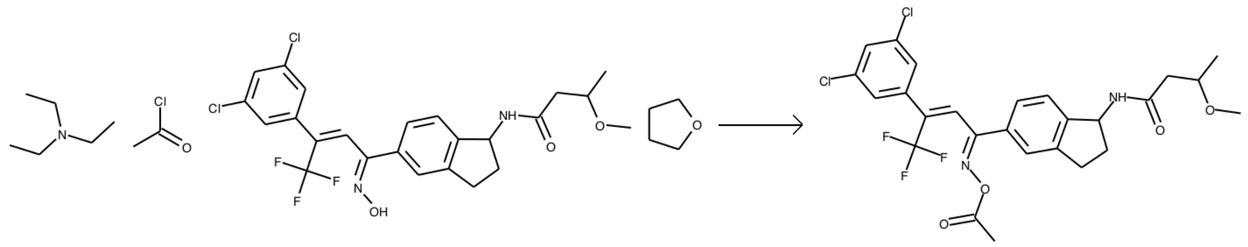

26.

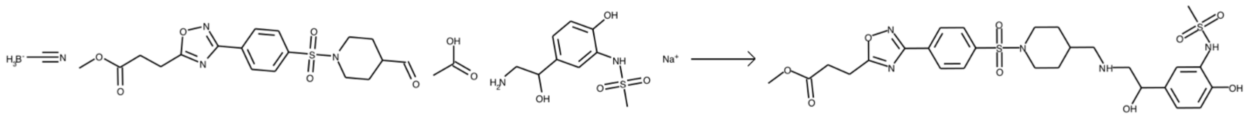

27.

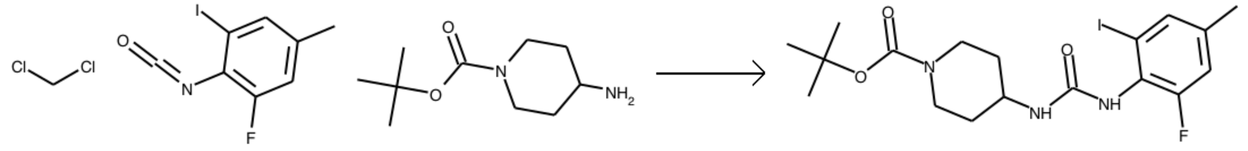

28.

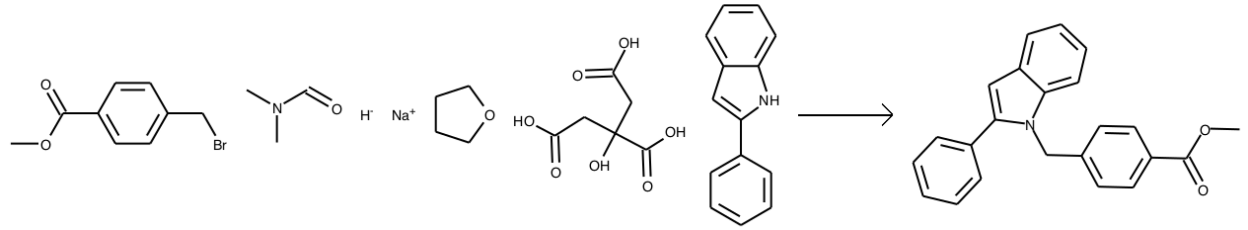

29.

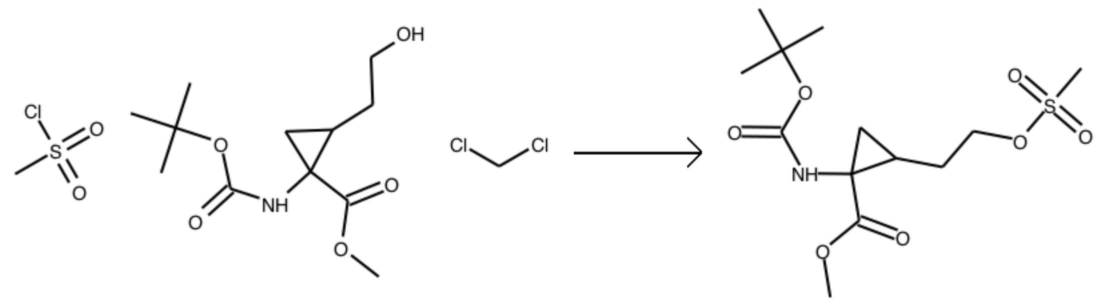

30.

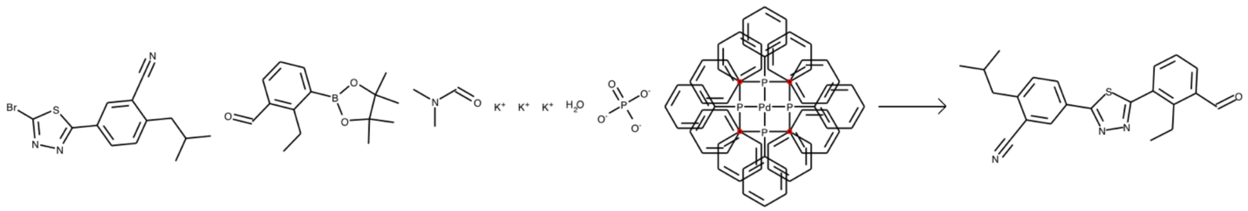

31.

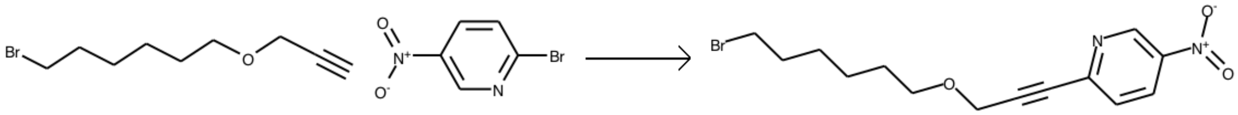

32.

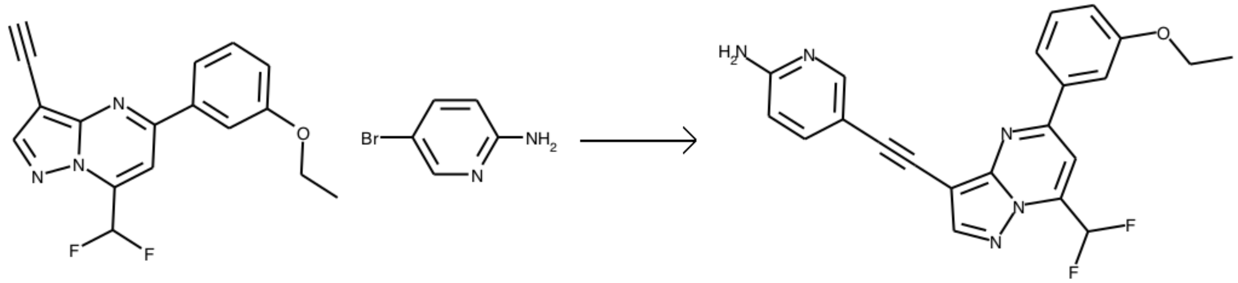

33.

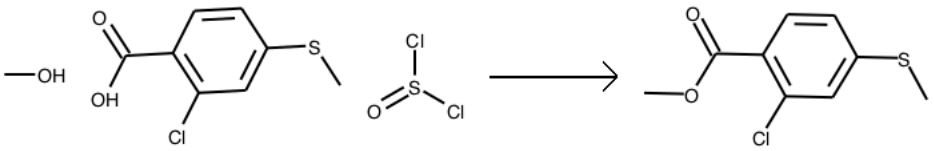

34.

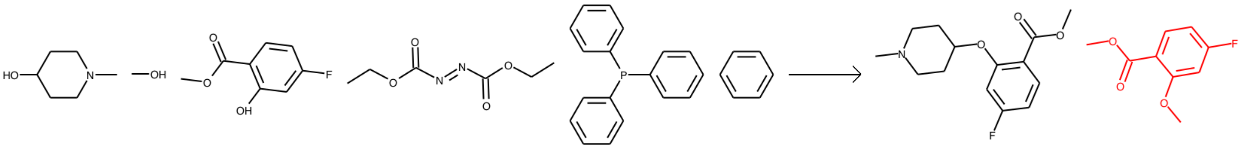

35.

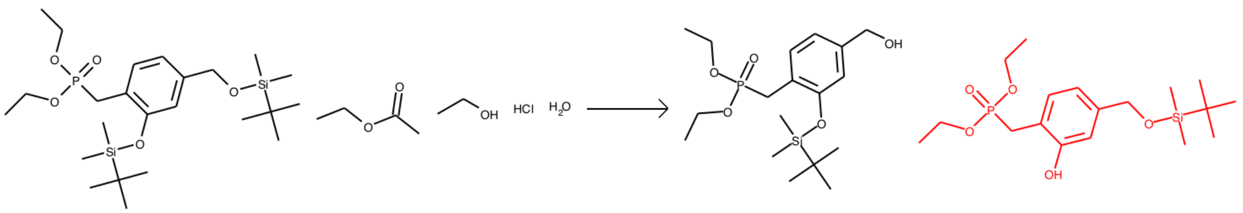

36.

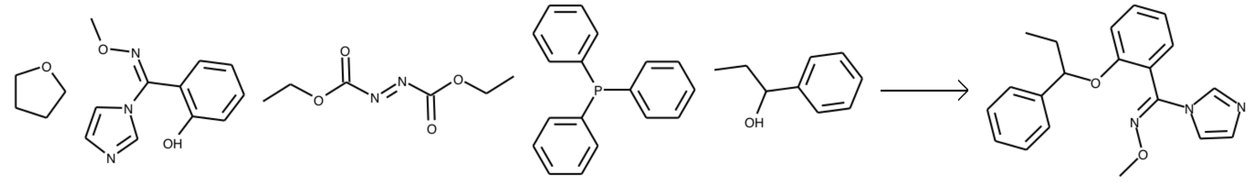

37.

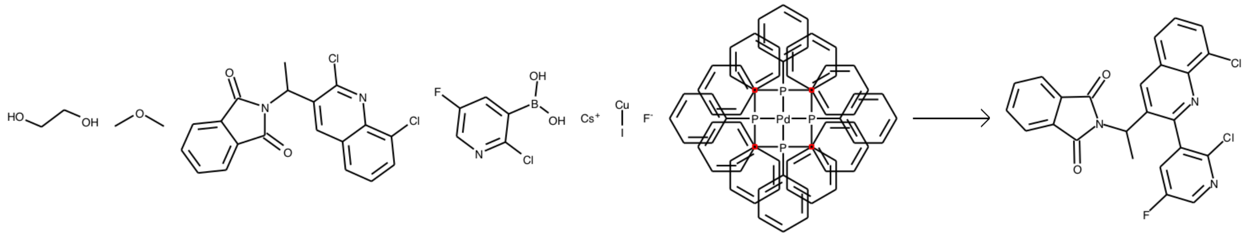

38.

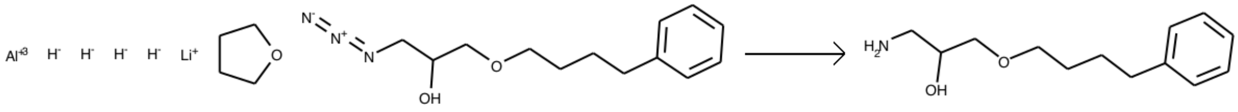

39.

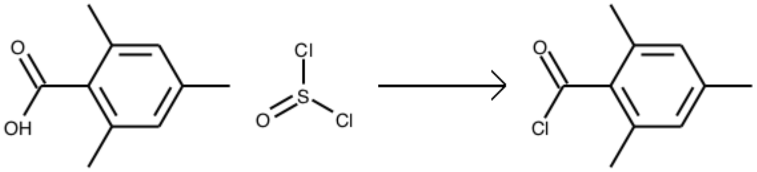

40.

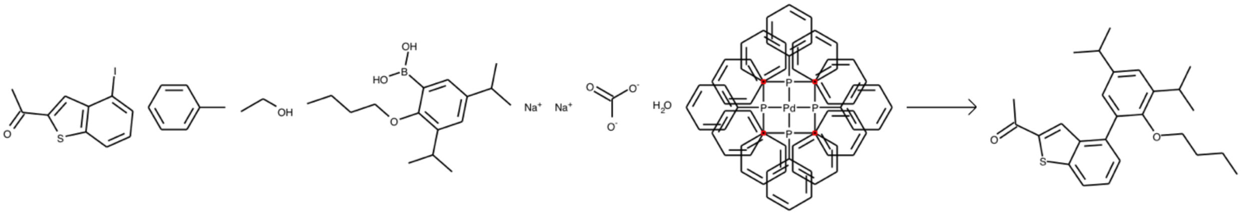

41.

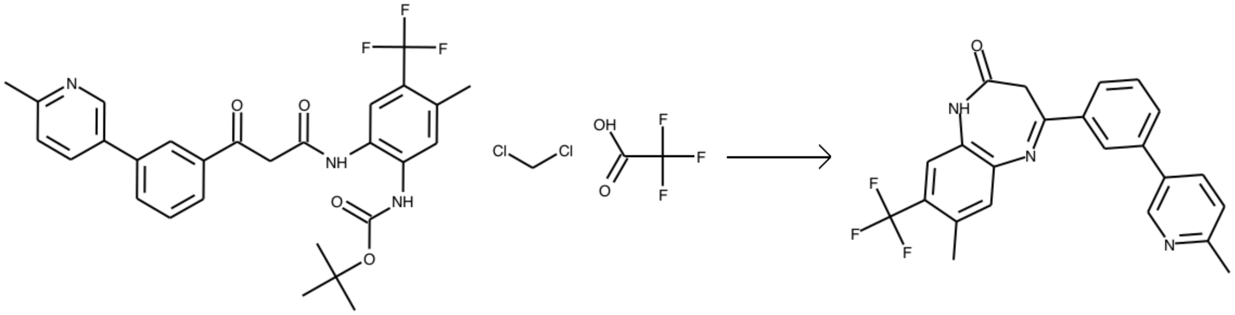

42.

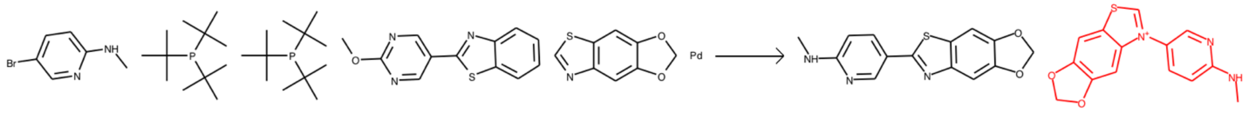

43.

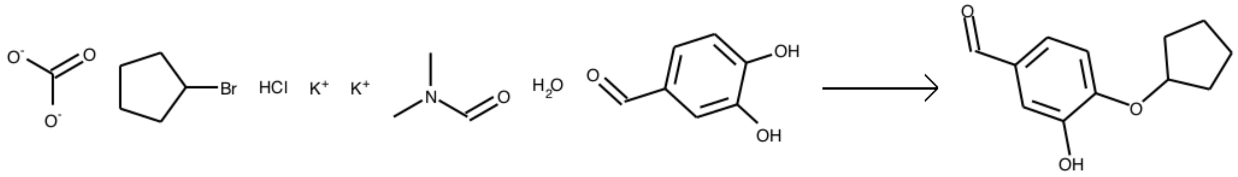

44.

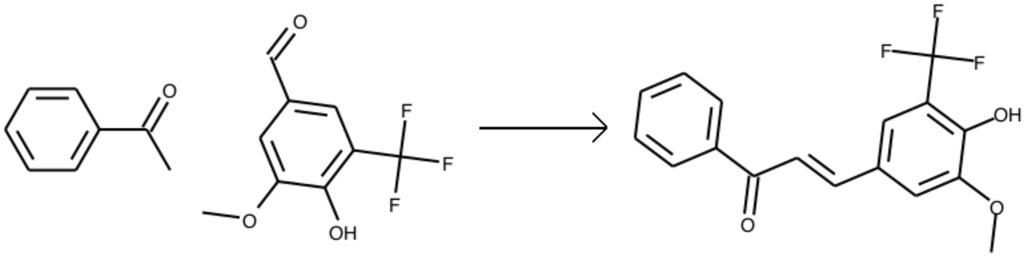

45.

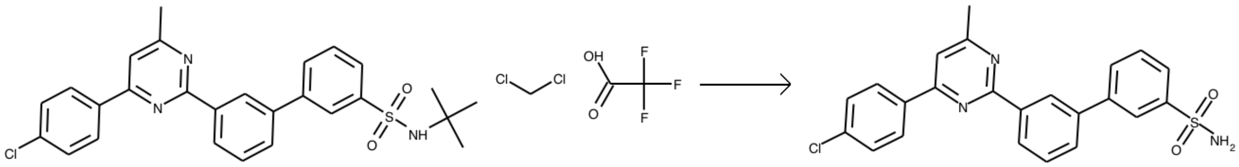

46.

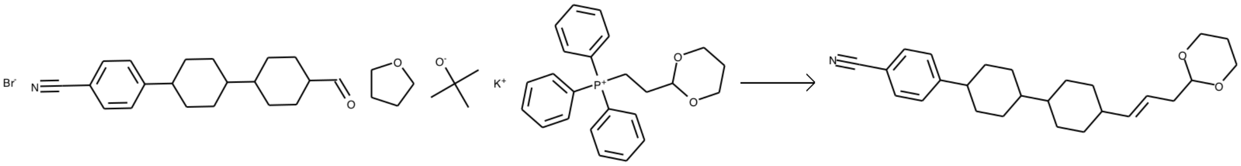

47.

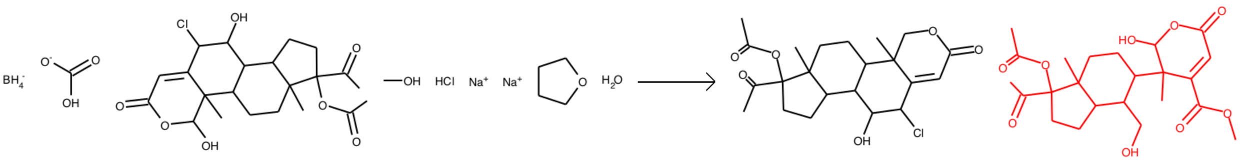

48.

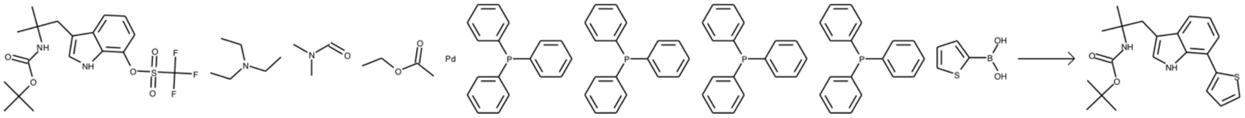

49.

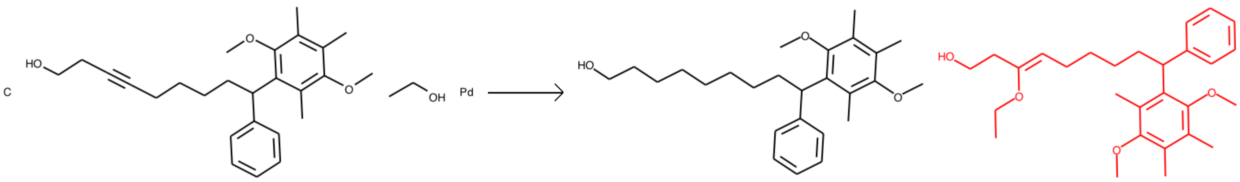

50.

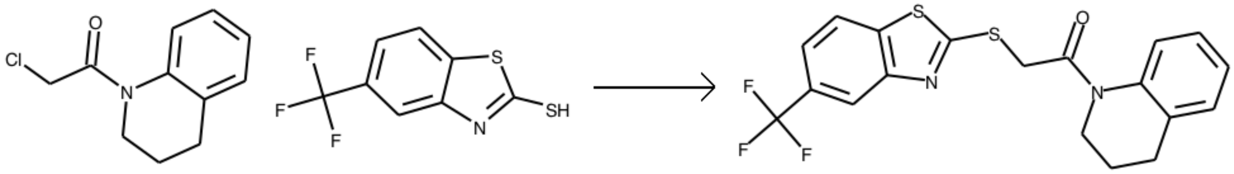

51.

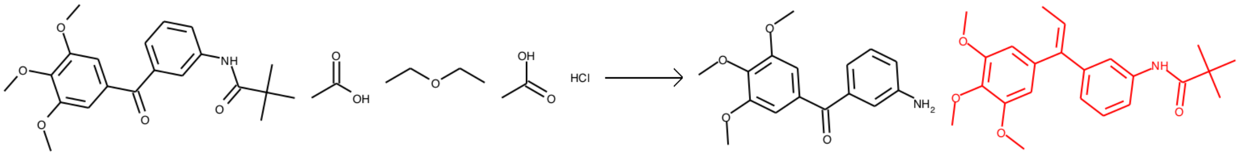

52.

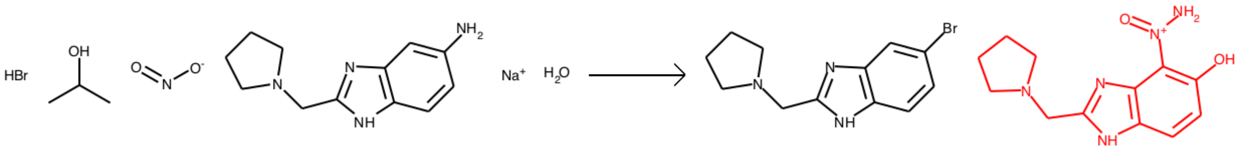

53.

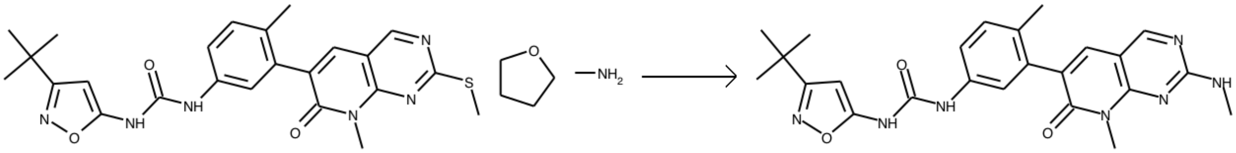

54.

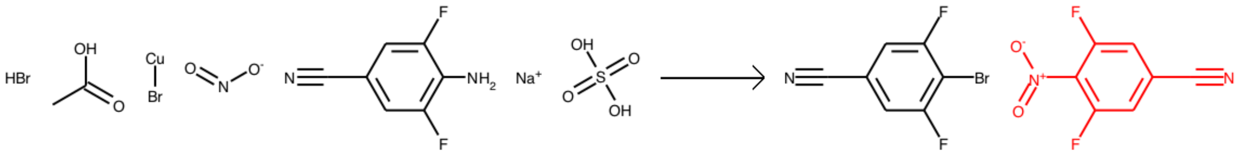

55.

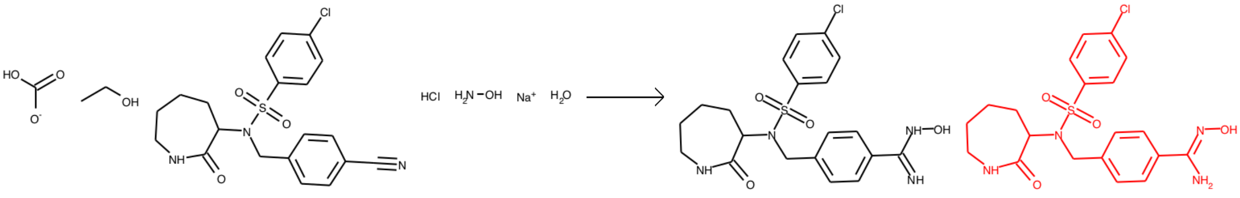

56.

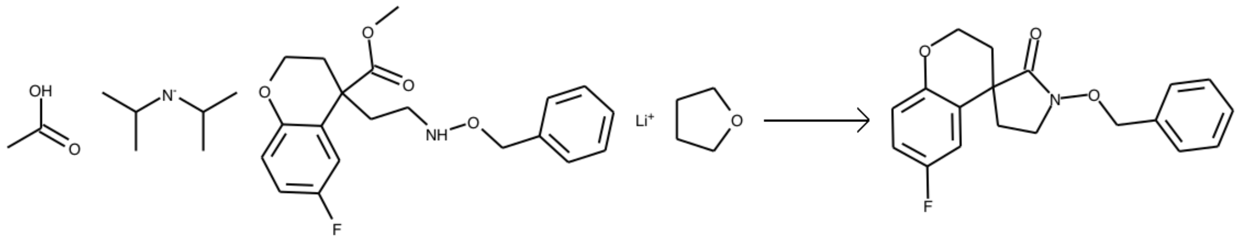

57.

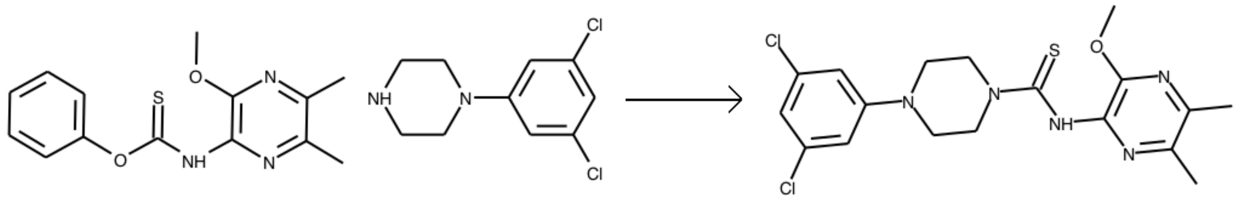

58.

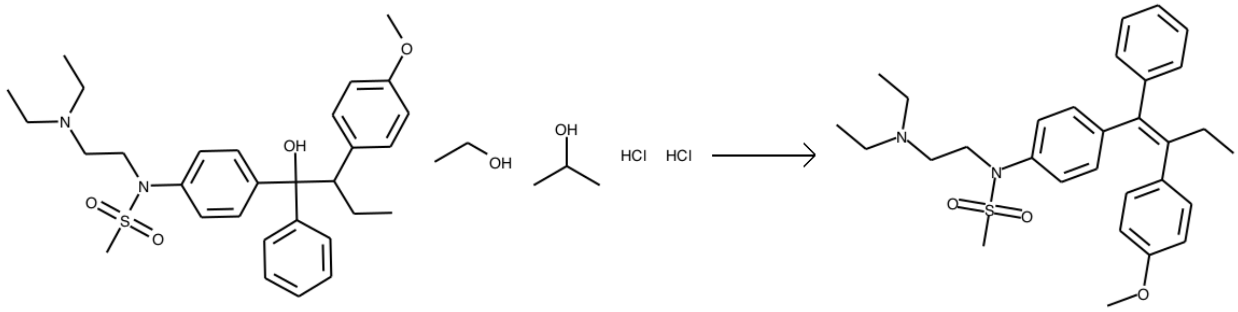

59.

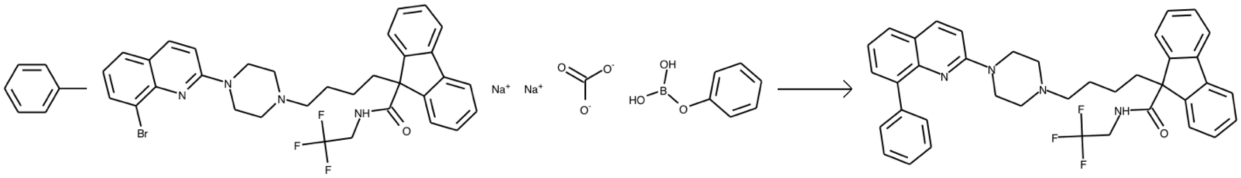

60.

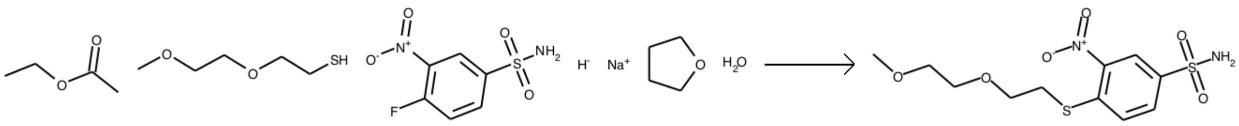

61.

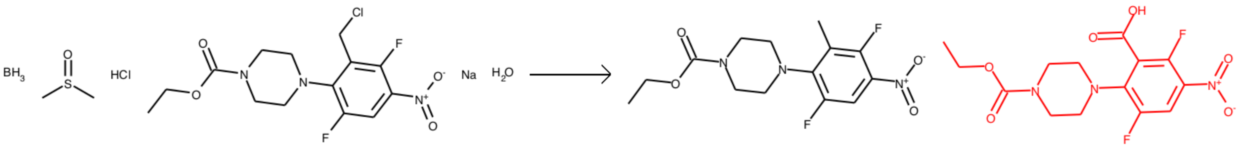

62.

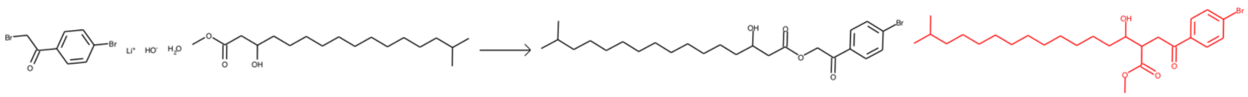

63.

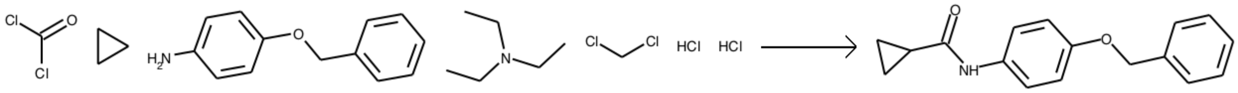

64.

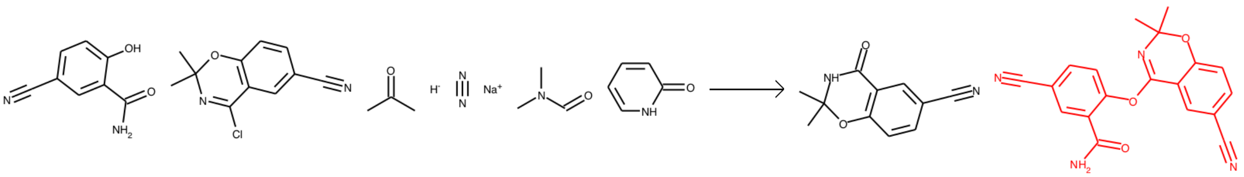

65.

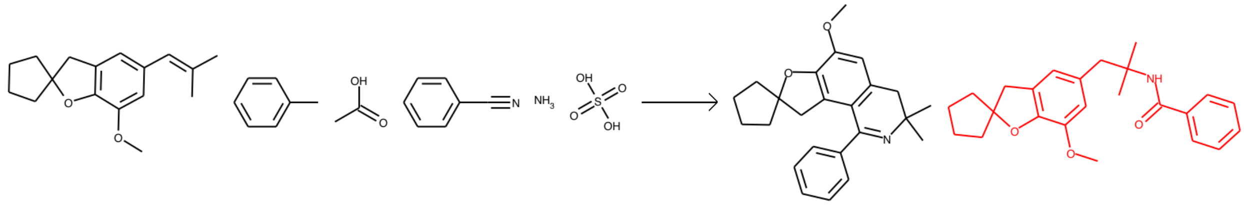

66.

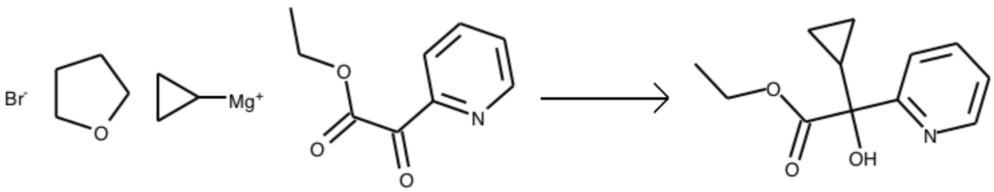

67.

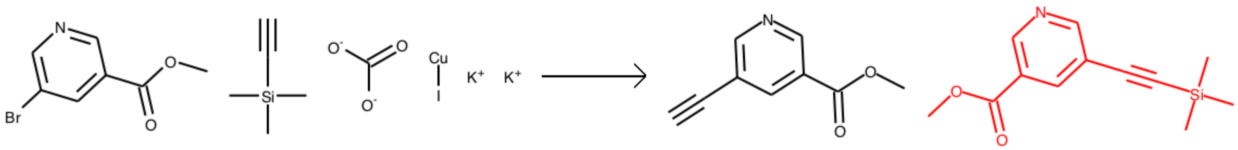

68.

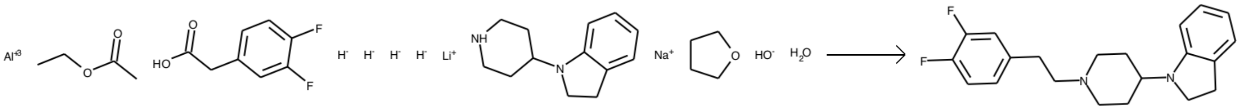

69.

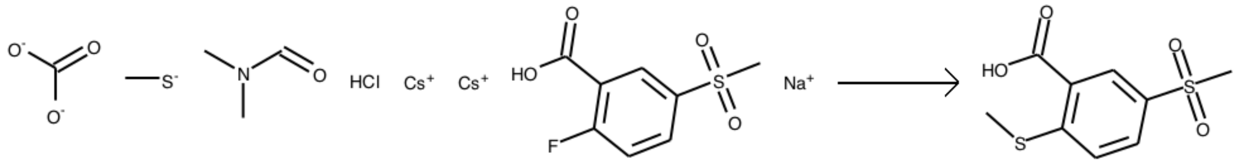

70.

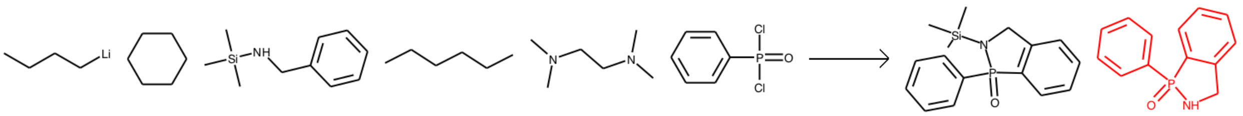

71.

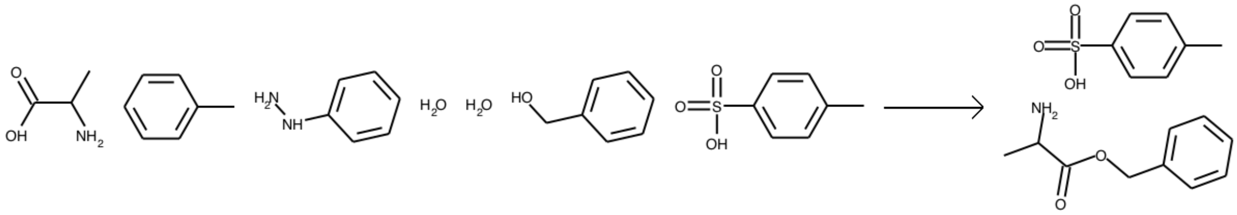

72.

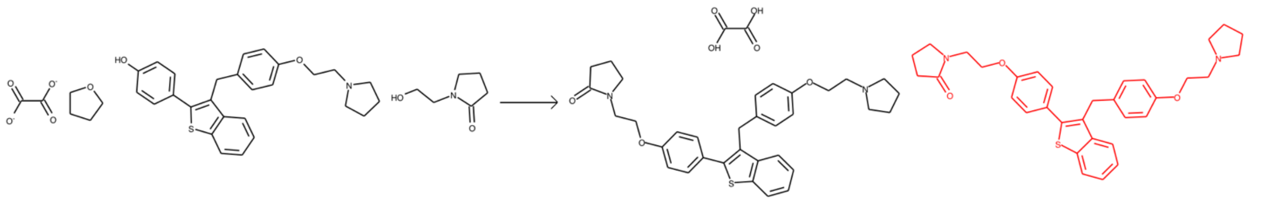

73.

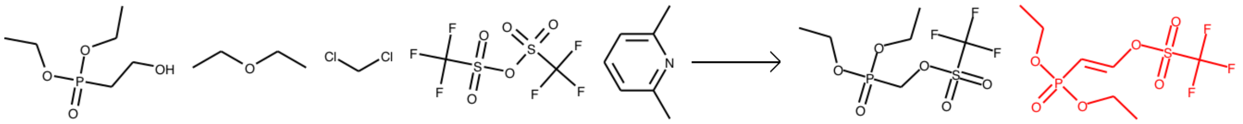

74.

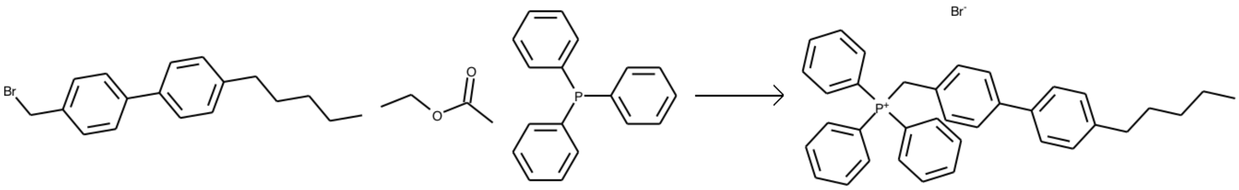

75.

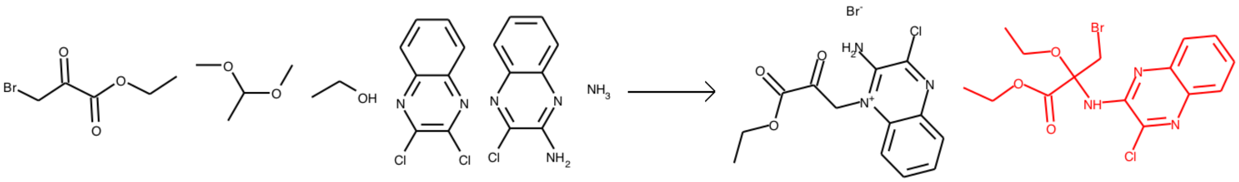

76.

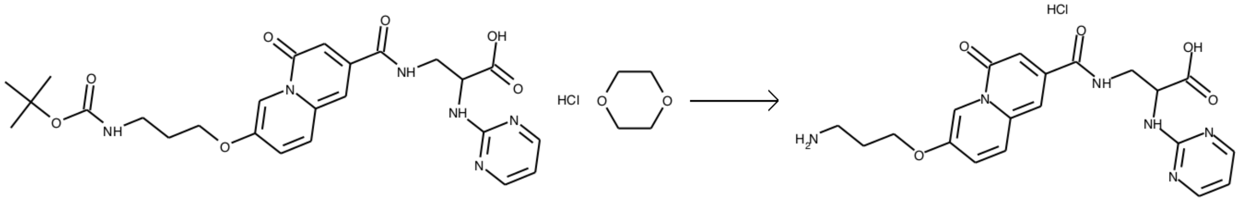

77.

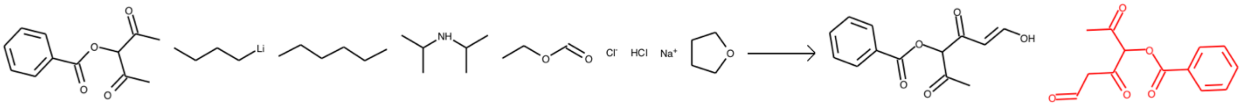

78.

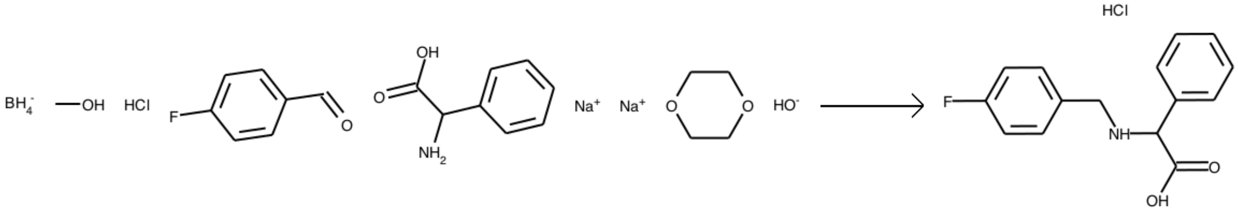

79.

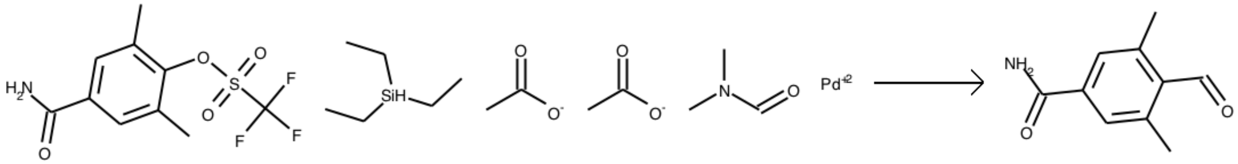

80.

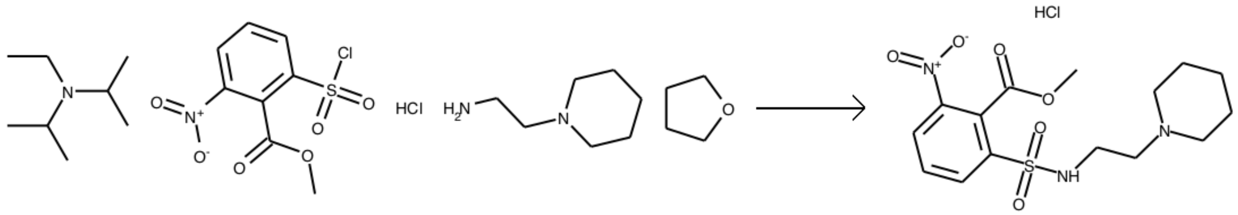

Supplement: Supplementary file 3 [file SC-010-C8SC04228D-s003.pdf]
